# Channel-Forming Bacterial Toxins in Biosensing and Macromolecule Delivery

**DOI:** 10.3390/toxins6082483

**Published:** 2014-08-21

**Authors:** Philip A. Gurnev, Ekaterina M. Nestorovich

**Affiliations:** 1 Physics Department, University of Massachusetts, Amherst, MA 01003, USA; E-Mail: gurnev@physics.umass.edu; 2 Program in Physical Biology, Eunice Kennedy Shriver National Institute of Child Health and Human Development, National Institutes of Health, Bethesda, MD 20008, USA; 3 Department of Biology, the Catholic University of America, Washington, DC 20064, USA

**Keywords:** gramicidin A, α-hemolysin, anthrax toxin, biosensing, stochastic sensing, ion channel, biological nanopore, protein translocation, targeted toxins, drug delivery, polymer transport

## Abstract

To intoxicate cells, pore-forming bacterial toxins are evolved to allow for the transmembrane traffic of different substrates, ranging from small inorganic ions to cell-specific polypeptides. Recent developments in single-channel electrical recordings, X-ray crystallography, protein engineering, and computational methods have generated a large body of knowledge about the basic principles of channel-mediated molecular transport. These discoveries provide a robust framework for expansion of the described principles and methods toward use of biological nanopores in the growing field of nanobiotechnology. This article, written for a special volume on “*Intracellular Traffic and Transport of Bacterial Protein Toxins*”, reviews the current state of applications of pore-forming bacterial toxins in small- and macromolecule-sensing, targeted cancer therapy, and drug delivery. We discuss the electrophysiological studies that explore molecular details of channel-facilitated protein and polymer transport across cellular membranes using both natural and foreign substrates. The review focuses on the structurally and functionally different bacterial toxins: gramicidin A of *Bacillus brevis*, α-hemolysin of *Staphylococcus aureus*, and binary toxin of *Bacillus anthracis*, which have found their “second life” in a variety of developing medical and technological applications.

## 1. Introduction: Channel-Forming Bacterial Toxins

During intoxication or internalization, many bacterial exotoxins form ion-conducting channels in membranes of the targeted cells or intracellular organelles. Channel formation is potentiated by a unique ability of many of these molecules to exist in two states: a stable water-soluble conformation and an integral membrane pore [1]. The resulting channels contribute to the toxin’s virulent action either directly or indirectly. Some channel-forming toxins directly kill compromised cells by inducing uncontrollable leaks of ions, water, and/or water-soluble metabolites [2,3,4]. The toxin-induced membrane perforation allows virulent cells to combat the host defense systems, to mediate escape of invasive bacterial cells into host cytoplasm from encapsulating phagosomes, to provide nutrients for bacteria, and to eliminate or control the competing bacterial cells [4,5,6,7,8]. Membrane-perforating bacterial toxins are often called pore-forming toxins (PFTs).

Distinctively different are the AB-type toxins that employ channel-forming parts for intracellular delivery of specialized catalytic subunits. In contrast to PFTs, AB-type toxins act in the cytosol, targeting specific intracellular substrates. The AB-type toxins are secreted as either single-chain proteins containing at least two functional domains, the receptor binding B domain and the active/enzymatic A domain, or as two (or three in the case of anthrax toxin) individual non-linked binary toxin subunits, an enzymatic/active A component and a binding/translocation B component [9]. For the AB-type toxin intracellular action, the enzymatic A component has to be delivered across the target cell membrane into the cytosol. Remarkably, the B components of the binary bacterial toxins secreted by several pathogenic species of *Bacillus* and *Clostridium* not only provide the binding site for the A components but also mediate the A component intracellular transport [9]. In particular, following the receptor mediated endocytosis, the B components form oligomeric transmembrane channels, that have been suggested to serve as active translocation pathways for the A component transport. The exact role of the B domain in transport of the single-chain AB-type toxins is debated; in particular, it is not always established whether the uptake of these toxins involves formation of ion channels. However, ion channel activity in planar bilayer membranes has been documented for at least two members of the family, namely diphtheria toxin, DT of *Corynebacterium diphtheriae* [10,11,12] and botulinum neurotoxin, BoNT of *Clostridium botulinum* [13,14]. Moreover, even though formation of ion channels in the artificial lipid bilayer systems has been reported for a number of binary bacterial toxin B components [15,16,17], the traditional pore-facilitated model of anthrax toxin internalization was recently challenged by an alternative “membrane rupturing” scenario [18].

This review is written to discuss several “classical” channel-forming bacterial toxins, which won their “second life” in a variety of developing biotechnological applications. Because the focus of this “Toxins” issue is on intracellular traffic and transport of bacterial protein toxins, we will mostly discuss the questions related to biosensing properties of the channel-forming bacterial toxins. More specifically, we highlight the applications where bacterial toxin pores were used to probe mechanisms of molecule detection and macromolecule transport. Remarkably, some of these applications, for instance those that develop the targeted toxin therapies to battle cancer, directly employ the unique ability of these proteins to intoxicate cells, whereas many other approaches use the channel-forming bacterial toxins as suitable tools able to respond to electrical, chemical or mechanical stimuli [19]. Note that the channel-forming proteins play quite distinct roles in the cell intoxication by PFTs and AB-type bacterial toxins. However, for the practical purpose of this review, we will use the term “channel-forming bacterial toxins” when referring to any of these types of toxins.

## 2. Biosensing and Polymer Translocation with Nanopores

The channel-forming toxins, and, more broadly, biological nanopores are molecules of immediate interest for biotechnology [19,20,21,22]. The ability of these molecules to generate selective and regulated pathways for water, ions, and water-soluble small and macromolecules in biological and artificial membranes offers exciting possibilities for a variety of medical and technological applications. To be directly examined for these applications, the biological nanopores are often incorporated into model lipid membranes where changes in the channel permeability to ions could be detected directly by applying transmembrane voltage and measuring ion current. The reconstitution methods range from the “classical” planar lipid bilayer and liposomes-based approaches to membranes supported on solid substrates and droplet interface bilayers (reviewed in refs. [19,23]). After gaining momentum from the pioneered studies by Kasianowicz and Bezrukov [24,25] where α-hemolysin (αHL) of *Staphylococcus aureus* was suggested to serve as a “nanoscopic cuvette” to study reaction dynamic some 20 years ago, the field rapidly exploded. Sensing with biological nanopores is based on reversible interruptions of ion current through an assembled pore in the model membrane that are generated by individual analytes, molecular complexes or nanoscale objects. Changes in this current resolved with uttermost precision using low noise feedback-loop operational amplifiers [26] could be used to determine presence, identity, structure, sequence, charged state, and kinetics of the studied objects or phenomena. In a way, this method is a molecular-scaled version of the resistive-pulse sensing technique suggested by William Coulter to count particles suspended in a fluid [27]. Within the reconstituted pore, single molecule binding affects the simultaneous concurrent transport of ~10^4^ s^−1^ of the small ions, thus providing great electrical amplification of the studied single-molecule process. Pores formed by the membrane-perforating toxins may act as elementary on/off “switches” activated in response to interaction with specific analyte molecules, and as sensors reporting on their membrane surrounding and providing a specific volume for the studied compounds to pass and/or to interact.

## 3. GrA, αHL, and PA_63_ as Nanopores of Choice in Biotechnology

Among a variety of channel-forming bacterial toxins potentially suitable for the biotechnological applications, one can highlight several dominant toxin molecules that, for a number of reasons, are in the lead of the field. Those are small peptide channel-forming gramicidin A of *Bacillus brevis*, which induces lesions in cell membranes; α-hemolysin of *Staphylococcus aureus*, a β-barrel heptameric pore-former, which also perforates cellular membranes; and binary toxin of *Bacillus anthracis*, in which the channel-forming component mediates delivery of the enzymatic subunits into target cell cytoplasm. The small channel-forming peptides, such as gramicidin A, can be chemically modified to serve as ideal on/off switches reacting to different stimuli and sensors of the bilayer membrane physical properties. The large β-barrel channels, such as α-hemolysin, are more suitable for the stochastic resistive-pulse sensing of the entering or passing small and macromolecules. The binary anthrax toxin is a unique tool to investigate the fundamental principles of protein translocation across the membranes and to reengineer the toxin’s properties for targeted killing of the cancer cells. In the following sections, we will first briefly review the biological functions of gramicidin A, α-hemolysin, and anthrax toxin and then discuss the main current and potential future applications of these three toxins in the growing field of biotechnology.

### 3.1. Gramicidin A of Bacillus Brevis

**Figure 1:**
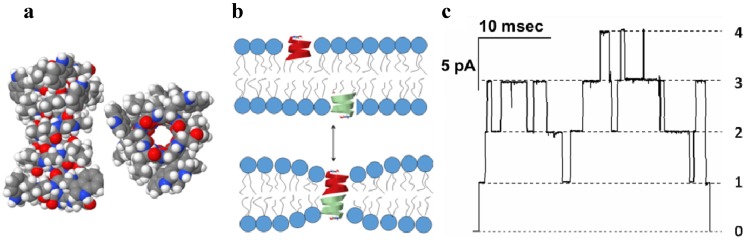
(**a**) Side and end views of the bilayer-spanning gramicidin A (GrA) channel. The energy-minimized structure represents a composite consistent with several NMR structures (PDB 1GRM, 1MAG); (**b**) GrA channels formed by the transbilayer dimerization of two β-helical subunits; (**c**) Single-channel current trace obtained with GrA in diphytanoylphosphatidylcholine bilayer [34]. Reprinted and modified with permission from reference [34].

Antibiotic gramicidin A (GrA) is secreted by soil bacteria *Bacillus brevis* in a mixture containing closely related peptide compounds [28]. GrA, which is a linear pentadecapeptide composed of 15 alternating L-D amino acids [29,30,31], is active against many gram-positive and several gram-negative bacteria [32]. Arguably one of the first ever described pore-formers, GrA generates defined and reproducible transmembrane water-filled channels [33] (Figure 1).

GrA membrane activity has been studied extensively over the last 40 years. Solution and solid-state NMR reveals a dimeric structure of the GrA pore where each molecular monomer adopts β-helical conformation and interacts with another one through hydrogen bonding between terminal formyl groups [35,36]. The luminal diameter of the pore that is formed by a peptide backbone is 4 Å, as determined from the channel’s permeability to alkali metal cations, H^+^, and water [37,38] (Figure 1a). Despite relatively low molecular weight of ~2 kDa, GrA creates well-defined and reproducible pores in planar lipid membranes (Figure 1c). The presented ion current recording, obtained under voltage-clamp experimental conditions, demonstrates sharp step-wise increments of the transmembrane current, where each upward “step” corresponds to formation of a GrA conductive dimer in a membrane, and the downward step indicates the dimer dissociation; maximum of four concurrently open GrA channels are seen in the record (numbered on the right side of Figure 1c). This type of single-channel recording allows for direct estimation of the major parameters of ion channel activity, current amplitude of the single pore and its average open time. These channels exhibit the structural and functional features typical for complex membrane proteins: defined conductive levels, sharp transitions between open and closed states (*i.e.*, spontaneous gating), and low excess electric noise of the pore open state. The gramicidin channels show an ideal selectivity for monovalent inorganic cations [38], that originates from the side chain dipoles of the peptide backbone facing the channel lumen [39,40]. The cation transport across the narrow GrA pore occurs in a single-file fashion through consecutive ion bindings to two specific sites in the pore [41].

The gramicidin channels are specifically interesting because they show a unique sensitivity to the properties of the surrounding lipid bilayer. The length of a pore-forming dimer is only ~26 Å, which roughly matches the thickness of the hydrophobic part of the lipid bilayer. Since the hydrophobic thickness of the gramicidin dimer is less than the thickness of the membrane [42,43], pore formation is almost always associated with a local membrane deformation [44,45] (Figure 1b). The energetic cost of that deformation is therefore a primary factor determining the channel’s functions. Because of that, the membrane lipid environment is a strong modulator of the GrA channel activity. Subtle changes in membrane properties such as membrane thickness, lipid composition, and ordered state influence parameters of GrA channels. Due to its relative simplicity, gramicidin has been recognized as unique ion channel for modeling and simulation of the fundamental principles of lipid/protein interactions, and membrane protein structure and function. The GrA channels are good candidates for the molecular sensing applications because of the relative simplicity of the peptide, defined characteristics of the ion channel conductance, and unique sensitivity of conductive parameters channel to the membrane structure [46,47]. In the developed applications, GrA is used to study mechanical properties [48] and charged state of membranes [34,49,50], to detect protein-membrane interactions [51], to probe enzyme activity [52] and protein-protein interactions, to create conductive components of bio-inspired diodes [53], and to fabricate components of drug delivery systems in nanomedicine [54].

### 3.2. α-Hemolysin of Staphylococcus Aureus

Alpha-hemolysin (αHL) is a water-soluble toxin secreted by the pathogenic bacterium *Staphylococcus aureus* [55,56]. Released as a 293-amino acid monomeric polypeptide with a molecular mass of 33 kDa, αHL is believed to bind to membrane protein receptors [57,58] and/or to specific lipids in the susceptible target cells [59,60]. Seven adsorbed monomeric subunits then associate and form a nonlytic prepore complex on the membrane. The subunits further penetrate the membrane to form a lytic pore [61,62]. Recent evidence suggests that the lytic pore formation may be not the only way of αHL toxicity. A specific toxin-induced activation of its putative metalloprotease receptor may trigger activation of intracellular cascades, leading to increased toxicity (for a review see reference [63]). However, the correct assembly of the αHL pores remains a necessary step for its toxic action [64].

**Figure 2:**
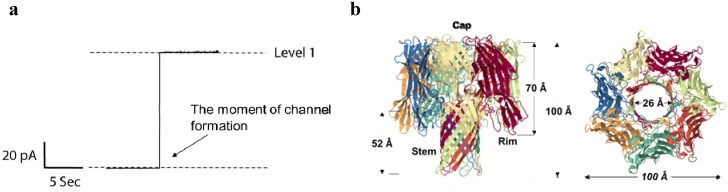
(**a**) Typical recording of a single αHL channel reconstituted into diphytanoyl-phosphatidylcholine membrane. Applied voltage is 100 mV. Channel current corresponds to ~100 pA in 1M KCl, pH 7.4; (**b**) Crystal structure of the αHL heptamer (top and side views are shown) (PDB 7AHL) [78]. The mushroom-shaped complex is approximately 100 Å tall and up to 100 Å in diameter, and the stem domain measures about 52 Å in height and 26 Å in diameter.

The first original study on αHL channel reconstitution in the model bilayers membranes was performed by Krasilnikov and co-workers over three decades ago [65]. Further electrophysiological studies refined our understanding of an αHL pore’s conductance, geometric, and gating properties [66,67,68,69,70,71,72,73,74,75,76,77]. The pore is weakly anion-selective at neutral pH [66] and a stable conductance of ~1 nS is observed in 1 M KCl solution at room temperature (Figure 2a).

Crystal structure of the αHL pore was solved in 1996 at 1.9-Å resolution [78] (Figure 2b). The pore heptamer has a hollow mushroom-like shape consisting of the “stem”, “cap”, and “rim” domains. The cap of the mushroom, which together with the rim forms the core of the protein complex, resides outside the target membrane and is formed by the N- and C-terminal ends of the monomers; it is composed of a β-sandwich and has a diameter of 100 Å. The stem part is a lytic transmembrane 14-stranded β-barrel assembled from seven β-hairpins, each contributed by an individual monomer. Two apparent constrictions with radii of 0.9 nm and 0.6–0.7 nm are located in the channel lumen, with the larger one being closer to the side of the cap. The interior of the β-barrel is primarily hydrophilic while the exterior has a hydrophobic surface. Molecular dynamic simulations were applied to estimate the diameter of the pore and the permeability of water through its side channels of the mushroom “head” [79]. By applying an electric potential across the pore, the authors were able to calculate the open-channel conductivity and the electrostatic potential along the pore length. Large pore dimensions and structural robustness of the αHL heptamer permit the wide usage of this bacterial toxin in a variety of developing biotechnological applications.

### 3.3. PA_63_ Component of Anthrax Toxin of Bacillus Anthracis

*Bacillus anthracis*, the bacterium that causes anthrax, is a large Gram-positive, rod-shaped, aerobic, spore-forming bacterial pathogen. The tripartite exotoxin (anthrax toxin) [80,81] and phagocytosis-inhibiting poly-D-glutamic acid capsule are the main virulence factors of *B. anthracis* [82]. The deliberate dissemination of *B. anthracis* spores in 2001 via the “anthrax letters” and their fatal consequences led to almost 13 years of continuing political and scientific efforts to develop medical countermeasures to protect humans from anthrax bioterrorism [83]. Because anthrax infections are infrequent, bacterial resistance of *B. anthracis*, even though described [84,85], is not a major focus of ongoing research. The recent efforts to disable the anthrax infection mostly focused on inhibiting the tripartite anthrax toxin (reviewed in [86]). Anthrax infection, especially in its inhalational form, is extremely difficult to treat because flu-like symptoms appear only after *B. anthracis* have multiplied inside the host and started to produce the anthrax exotoxin [87,88]. At this stage, the aggressive antibacterial therapy can inhibit the bacterium growth, but the infection can still be lethal because of the accumulation of the toxin [89]. The recent progress made in understanding of anthrax toxin structure, arrangement, cellular uptake and functions is significant (reviewed in refs. [90,91,92,93,94,95,96,97,98,99,100]). Briefly, the binary anthrax toxin consists of two enzymatically active A moieties: lethal factor (LF) and edema factor (EF), and a single shared binding and translocation moiety: protective antigen (PA). The term “protective antigen” originates from the ability of this protein to stimulate production of the protective antibodies when used in anthrax vaccines. Individually, anthrax toxin’s PA, LF and EF components are nontoxic; however, PA combinations with lethal toxin (LT = LF + PA) or with edema toxin (ET = EF + PA) are primarily responsible for the anthrax symptoms and lethality.

**Figure 3:**
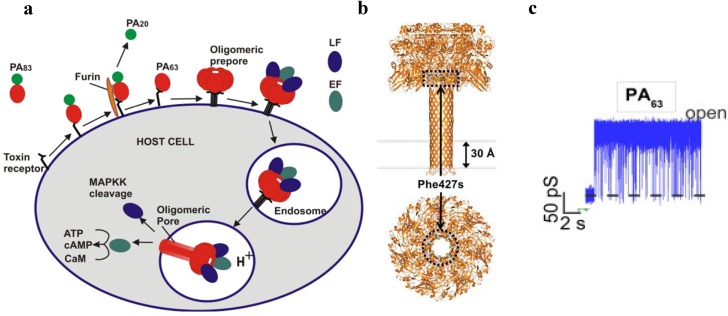
(**a**) A schematic model of *Bacillus anthracis* toxins cell entry; (**b**) Side and top views of anthrax toxin PA_63_ component (the symmetric model, PDB:1V36) [101]. The Phe427s are marked because of their importance in the transport properties; (**c**) Conductance of a single PA_63_ channel reconstituted into planar lipid membranes demonstrate fast flickering between open and closed states at 1-ms time resolution [102]. Reprinted with permission from references [86,101,102]. Copyright 2011 Wiley, 2012 Elsevier.

The internalization process of anthrax toxin involves several stages (Figure 3a).

First, full length PA_83_ binds to the CMG2 and TEM8 receptors [103,104] on a host cell surface and is subsequently cleaved by extracellular furin protease to PA_63_ [105]. PA_63_ then undergoes oligomerization, which leads to formation of heptameric, (PA_63_)_7_ [106] and octameric, (PA_63_)_8_ [107] ring-shaped prepores. The formation of (PA_63_)_7_ and (PA_63_)_8_ generates, correspondingly, three [108] and four [107] LF/EF binding sites at the interface of two adjacent PA_63_ molecules and causes receptor-mediated endocytosis of the anthrax toxin tripartite complexes [109]. The oligomeric PA_63_ prepore then undergoes significant structural changes promoted by the acidic endosomal environment, which results in the formation of a mushroom-shaped ion channel (125 Å in diameter with 70 Å long cap, and 100 Å long stem [110]) (Figure 3b), preferentially selective to cations [15]. When incorporated into the bilayer lipid membranes, PA_63_ was reported to form ion-permeable cation-selective ion channels [15,111,112] prone to voltage-dependent closures typical for the other β-barrel channels. The characteristic property of PA_63_ single channels as well as the channel-forming components of binary clostridial C2 and iota toxins reconstituted into bilayer membranes is the non-voltage-dependent flickering of ion current between the open and completely closed states [16,17,113,114,115] (Figure 3c). Interestingly, the current noise of the F427A (ϕ-clamp) mutant (see Section 5.2) of PA_63_ is mostly free of this complex behavior [115]. A PA oligomer then is believed to act as an effective translocase, which unfolds and translocates LF and EF inside the cell using the proton gradient across the endosomal membrane (pH_endosome_ < pH_cytosol_) [116,117,118,119,120,121]. In an alternative model, the anthrax toxin catalyzes rupture of the endosomal membrane and toxic complexes are released into the cytosol [18]. Once in the cytosol, LF and EF actuate their catalytic actions. LF is a Zn-metalloprotease that cleaves mitogen-activated protein kinase kinases [122,123] and NLrp1 [124], resulting in disturbing host cell signal transduction. EF is a calmodulin-dependent adenylyl cyclase, which increases the cAMP level in the cells, contributing to dissemination of *Bacillus anthracis* in the host [125]. The key tissue targets responsible for the poisonous effect of LT and ET were recently identified [126]. LT and ET were shown to target the cardiovascular system and liver, respectively.

Anthrax toxin represents a unique arrangement of proteins where intracellular delivery of the enzymatic components is governed by the channel-forming component. Therefore, it provides an exceptional biological tool to study molecular details of protein transport and to rearm this toxin against malignant cells.

## 4. Channel Forming Bacterial Toxins for Molecular Sensing

### 4.1. Probing Structure, Charge and Physical State of the Membranes with GrA

The physical properties of biological membranes, namely membrane composition, lipid content, and asymmetry in lipid distribution between the bilayer leaflets and prevalence of the charged species are among the general factors affecting functions of membrane-embedded proteins. Apart from the specific protein/lipid interactions, the effect of the colligative membrane properties such as bilayer thickness, viscosity, bending rigidity, and the so-called intrinsic curvature [127] on membrane protein functioning is not well understood. The need for molecular probes is well fulfilled by small channel-forming peptide-based toxins, such as gramicidin A, studied in a model systems under controlled conditions. Originally, GrA and its derivatives were used to study membrane mechanics. Andersen and colleagues investigated the correlations between membrane thickness, viscosity, and bending rigidity, and channel lifetime. For details of these studies, we direct the reader to several reviews [48,128].

Apart from their well-recognized application in studying membrane mechanics, small channel-forming peptides have been employed as sensitive probes to monitor surface membrane properties, in particular, membrane electrostatics. For the most part, the phospholipid molecules, which are the building blocks of the cellular membranes, contain zwitterionic or negatively charged headgroups exposed on the membrane/water interface. Distribution of these groups affects combined operation of the membrane-associated molecules, because electrostatic forces are important for many interactions within and between macromolecules. The interaction of proteins, nucleic acids, phospholipids, and their supra-molecular assemblies with the membranes affects general system electrostatics. In addition, the membrane surface potential is involved in regulation of membrane transport and cell/cell recognition [129,130]. The small channel-forming peptides can serve as the sensitive probes to monitor the properties of their immediate membrane surrounding.

It was established relatively early that the conductance of GrA channels is a strong function of the membrane surface charge [131]. The ion conductance of the bilayer-embedded GrA pore increases with an increase in percentage of the negatively charged surface bilayer groups. The charges on a membrane surface attract counter-ions and reduce the concentration of the co-ions near the entrance of the pore, thus increasing or decreasing a number of the cations, depending on the sign of the surface charge. The effects of the surface charge in GrA conductance are especially strong at low electrolyte concentrations.

**Figure 4:**
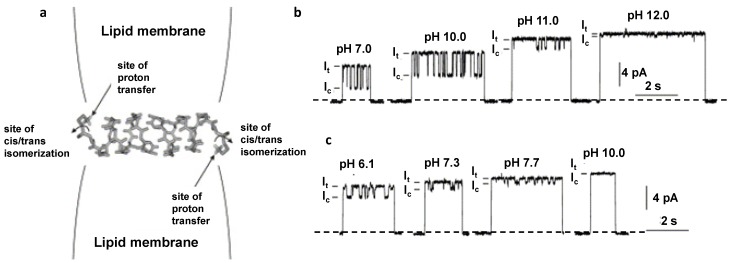
(**a**) Model representation of the engineered GrA-ethylenediamine channel in a lipid bilayer [49]. At both entrances of the channels, an ionizable site (the primary amino group in gram-ethylenediamine; the imidazole group in gramicidin-histamine) is connected to the gramicidin backbone via a carbamate linkage). Representative single-channel currents of gramicidin-ethylenediamine (**b**) and gramicidin-histamine (**c**) at different pH values [49]. The closed channel states are seen as the zero current levels. The currents of the *trans* (I_t_) and *cis* (I_c_) isomeric states of the channel are indicated. Reprinted and modified with permission from reference [49]. ScienceDirect open archive.

Rostovtseva and colleagues analyzed the gramicidin pore conductance varying percentage of the charged lipids in a bilayer composition at different pH and salt concentrations [50]. Deviations of a single pore conductance from the predictions made using the Gouy-Chapman formalism allowed for estimation of the “intrinsic” pK values of the charged lipids at the surface of the bilayer. Thus, the GrA pore conductance was used to detect minute changes in the membrane surface charge density, supporting the results obtained using different surface charge identifying techniques [129,132]. Borisenko and collaborators [49] attached protonated chemical groups on the gramicidin molecules to create gramicidin-ethylenediamine and gramicidin-histamine peptides (Figure 4a).

Both GrA variants were able to form single-molecule pH-sensitive pores, by displaying additional current flickering in response to the membrane surface acidity changes (Figure 4b,c). Similarly, modified GrA channels with lysine substitution on the C-terminal, allowing for channel activation at the specifically high pH were recently reported [133].

**Figure 5:**
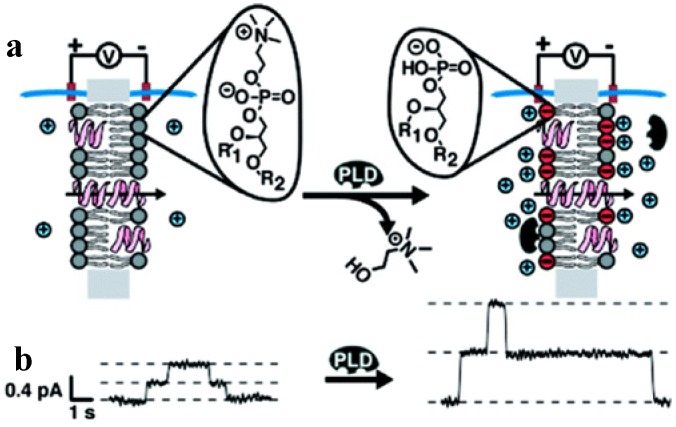
Monitoring activity of phospholipase D (PLD) in the model lipid bilayers by changes in single channel conductance of GrA pores [52]. (**a**) As PLD hydrolyzes electrically neutral PC lipids and produces negatively charged PA lipids, the membrane negative charge is generated. It induces accumulation of the cations close to the membrane surface, leading to a significant increase in channel conductance. Negative charges are shown in red, and positive ions are shown in blue; (**b**) GrA unitary current *vs.* time recordings before and after addition of PLD. Reprinted with permission from reference [52]. Copyright 2009 American Chemical Society.

Sensitivity of the gramicidin channels to the membrane surface properties determined their use in the applications focusing on probing changes in the membrane charged state. Mayer and colleagues [52] used the unmodified GrA to monitor enzymatic activity of the phospholipases D and C (PLD and PLC). These enzymes are cleaving specific phosphodiester bonds in the phospholipid headgroups, resulting in accumulation of charged or non-charged lipid species. Charged state of the membrane surface was changed, which, in the presence of the low electrolyte solution, was expected to influence GrA channel conductance. Indeed, application of PLD to the uncharged phosphatidylcholine membranes significantly increased the unitary GrA channel conductance. This change could be related to a conversion of the phosphatidylcholine lipids into the negatively charged phosphatidic acid, which, in turn, generates an increase in cation concentration at the membrane surface and, thereby, inside the channel (Figure 5).

PLC activity in the negatively charged bilayers produces a significant decrease in the GrA conductance, reducing membrane content of the negatively charged phosphatidylinositol. Similarly, picomolar additions of alkaline phosphatase were monitored by attaching an enzyme-cleavable phosphate group on the C-terminal end of the GrA derivative [134]. In this case, cleavage of the negatively charged phosphate group reduces the concentration of cations only in the direct vicinity of the channel opening. 

GrA and similar small toxins can be used to probe the electrostatic interactions of charged peptides and counter-ions with the membranes. For example, the membrane surface charge modification induced by adsorption of polyvalent cations and positively charged cargo peptides could be quantitatively detected [34,51]. Similarly, conductance of another small toxin, syringomycin E [135], was used to monitor adsorption of DNA on the positively charged bilayers [136].

### 4.2. Molecular Sensing with αHL, PA_63_, and GrA

Accurate and rapid molecular detection is vital for basic research, medicine, technology, and defense. Biological nanopores, including the channel-forming bacterial toxins, are natural single-molecule biosensors evolved to stochastically detect their substrates. In the model lipid bilayers, this detection is often manifested by reversible current fluctuations between open and closed states. The frequency, duration and magnitude of the “on/off” closures are defined by the concentration, size, charge, and other physicochemical characteristics of the substrates and analytes.

Over the past two decades, wild-type and genetically or chemically modified αHL was extensively investigated as the molecular sensor able to detect a variety of analytes. The ionic species and organic molecules tested include DNA and RNA biopolymers [137], proteins [138], cyclodextrins [73,139,140], divalent metal ions [141,142,143], phosphate anions [144], trinitrotoluene [145], styryl dyes [146], β-lactam antibiotics [147,148], heparin [149], chemical warfare agents [150], and neurotransmitters [151].

**Figure 6:**
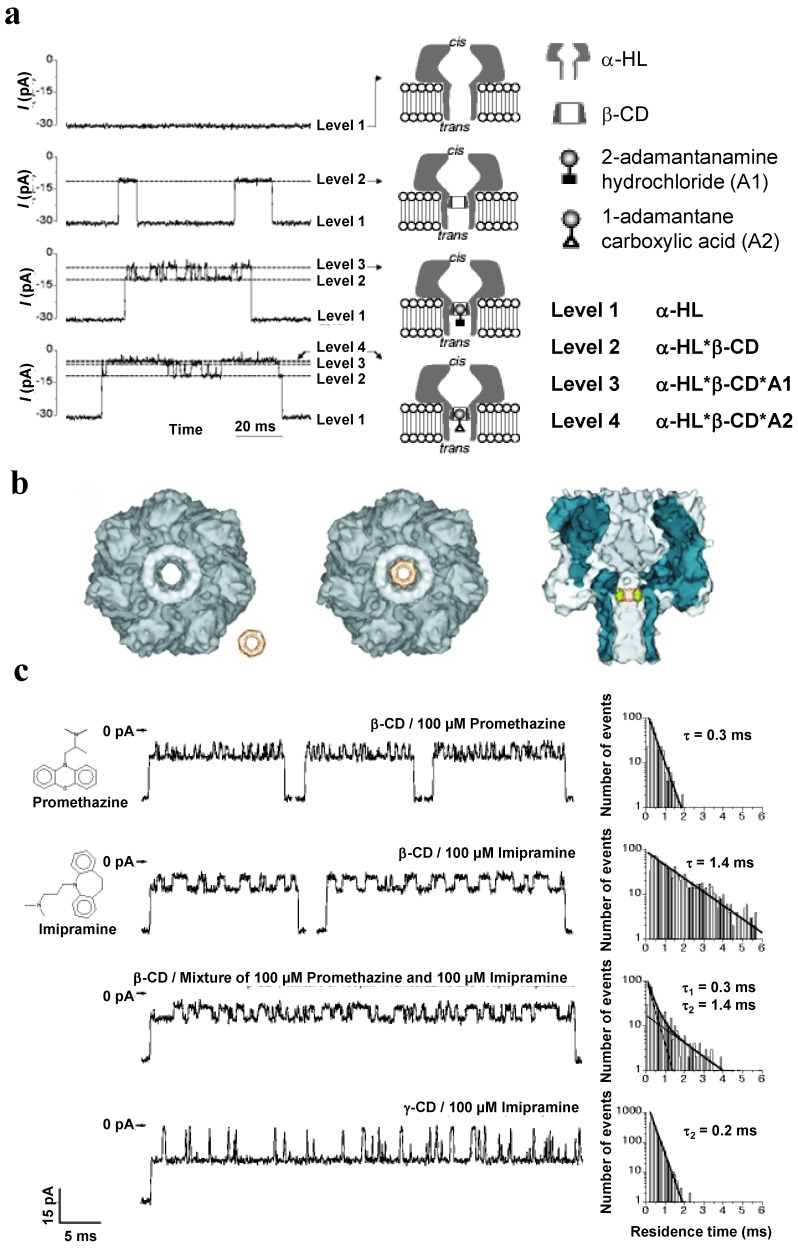
(**a**) Bilayer recordings showing the interaction of a single αHL pore with β-cyclodextrin and model analytes 2-adamantanamine and 1-adamantanecarboxylic acid; (**b**) Molecular graphics representation of the interaction between αHL and cyclodextrin; (**c**) Analysis of drug molecules by stochastic sensing with αHL and a βCD or γCD adapter. Reprinted and modified with permission from reference [139]. Copyright 1999 Nature Publishing Group.

One of the fascinating examples of the αHL’s biosensing properties is its ability to reversibly bind β-cyclodextrin-based (βCD) molecular adapters. In 1999, Bayley and colleagues equipped a single αHL channel with an internal, non-covalently bound βCD that was able to mediate channel blocking by organic analytes, such as adamantanamine hydrochloride, adamantine carboxylic acid, promethazine, and imipramine (Figure 6) [139]. The symmetry match between the heptameric αHL channel and the 7-fold-symmetrical ring-shaped βCD as well as a hydrophilic β-CD’s exterior and proper dimensions assure comfortable fit between the single βCD and the αHL channel lumen (Figure 6b).

The blockage events were detected when the non-modified cyclodextrins were added to the *trans* but not *cis* side of the chamber. Note that 6-fold symmetrical αCD and 8-fold symmetrical γCD were also able to reversibly interact with the channel [139]; αHL mutants able to bind βCD from the *cis* side were later identified [73]. Because the βCD-induced current blockage events were partial (64% of the total current) (Figure 6a, 2nd trace), leaving possibilities for the further current decrease, and because of the known ability of cyclodextrins to host different organic “guest” molecules in their hydrophobic interior, the αHL/βCD system was suggested [139] and further investigated as a stochastic sensor for organic molecules. It turned out that several βCD analytes (Figure 6a, 2nd and 3rd traces) were able to further decrease the current through a single αHL channel, while not affecting the completely open channel. The recorded signals not only were used for the analyte detection but also for their quantification. Moreover, the authors explicitly showed that, when applied in mixtures, the αHL/βCD “guests” could be stochastically detected and quantified simultaneously by a single sensor element (Figure 6c) [139]. Interestingly, the wild type αHL and various mutants became more anion selective after the βCD binding, whereas the negatively charged adapter, hepta-6-sulfato-βCD generated selectivity to cations [140]. To understand how βCD affects ion selectivity, Roux and colleagues performed molecular dynamic simulations and potential of mean force calculations using αHL with and without the βCD adapter [152,153]. The authors demonstrated that the narrow βCD adapter increases the anion selectivity of αHL because it locally reduces the radius of the pore, which results in a decrease in ionic screening and dielectric shielding of the strong electrostatic field induced by a ring of positively charged side chains of αHL [153]. βCD also induces a partial dissolvation of ions and affects the orientation of nearby charged residues [152].

Interaction of βCD with αHL was shown to be pH and voltage dependent; the apparent βCD dissociation constant varied from ~3.1 × 10^−3^ to ~2.1 × 10^−2^ M in pH 5–11 range, and from ~3.1 × 10^−3^ to ~1.4 × 10^−2^ M with voltage change from −40 mV to +40 mV [73]. Moreover, the αHL mutants that bind the βCD adapter ~10^4^ times more tightly than the wild type have been designed and investigated both with the planar lipid bilayers and high resolution x-ray crystallography [154]. Clearly, the mutant pores with the extended βCD dwell time can serve as improved noncovalent molecular adapters [155], because sensing with molecular adapters would be more effective if the adapter did not frequently dissociate from the channel, leaving αHL unable to detect analytes [156]. To increase strength of the βCD/αHL interaction, the authors covalently attached β-CD within the α-HL pore in two possible molecular orientations by using a specific linker [156]. A modified βCD-based adapter, where a single oxygen atom in its ring skeleton was replaced by a disulfide unit, was also investigated [157]. A heptameric αHL pore capable to accommodate two different βCD-based adapters at distinct binding sites was engineered [158]. Non-modified β-CD and hepta-6-sulfato-βCD were lodged simultaneously within the lumen of a single αHL pore that was genetically engineered to accommodate them. The space between the adapters was characterized as ~4400 Å^3^ “nanocavity” for which the adapters served as gates at the *cis* and *trans* ends of the cavity [158]. The γCD-based adapters were employed to investigate the dynamic aspects of the Hofmeister effect [159] and to discriminate between the S- and R-enantiomers of the ibuprofen and thalidomide drug molecules [160].

Cyclodextrin’s ability to enter a protein pore and reversibly occlude it was explored by a rational design of the channel blocking antitoxins to target both αHL [161,162] and binary bacterial anthrax, C2, and iota toxins [102,114,163,164,165,166,167,168,169,170] (recently reviewed in refs. [86,115,171]). Briefly, the idea was to directly obstruct these channels from the physiologically relevant *cis*-side by compounds of the same symmetry as the target pore [163]. According to the diffusion model of Berezhkovskii and Bezrukov, for the blocking molecule to be therapeutically effective the attraction between the molecule and the channel should be strong enough to allow the molecule to stay in the channel for sufficiently long, thus blocking translocation of other molecules [172,173]. The PA_63_ prepore internal diameter was estimated to be between 20 and 35 Å [106] with the PA_63_ pore’s constriction region not exceeding 12 Å [113,174,175]. This finding guided the choice of the ~15-Å 7-fold symmetrical βCDs as the potential core structures for blocker design. Because the PA_63_ channel, the binding component of the anthrax toxin is preferentially selective to cations, βCD molecules were rationally modified to incorporate seven positive charges (amino groups) covalently linked to the cyclodextrin’s core by hydrocarbon linkers of different nature and length [163]. Several 7+βCDs were custom-synthesized and tested in the planar bilayers, in cell assays [102,114,163,168], and *in vivo* [167]. When a single phenyl group was introduced into each of the seven hydrocarbon linkers, the βCD blocker affinity to the pore was enhanced more than 10 times [102,165]. It was suggested that this difference is defined by the additional stabilizing interactions between the introduced aromatic groups and ϕ-clamp (see Section 5.2). Indeed, two of the tested βCD blockers were significantly less potent with the F427A mutant of PA_63_ [102]. The authors also made a step towards understanding the physical forces responsible for the pore-blocker interactions by analyzing the dependence of interaction strength and underlying kinetics on the salt concentration and applied transmembrane potential [102]. It was shown that in the binding reaction of the 7+βCD to the channels, salt-concentration independent, short-range forces predominate. In the case of the blockers equipped with the aromatic linkers, the binding was further enhanced. At moderate and low salt concentrations, the blockers’ residence times were influenced by long-range Coulomb interactions. The increase in the residence time as a function of transmembrane voltage showed the existence of an additional electrostatic component in the blocker/pore interactions.

Aside from 7+βCDs, PA_63_ is permeable to a number of small-molecule cationic blockers [111,118,174,175,176] and some of them, especially those carrying the aromatic fragments [118] bind the channel at the low nM concentrations. It would be interesting, apart from the therapeutics applications, to explore the ability of the channel to detect positively charged analytes for the biosensing. In a different application, planar lipid bilayer measurements with anthrax toxin components were investigated as a rapid quantitative tool to recognize specific proteins and to screen for anthrax therapeutics [177]. The authors showed that the full-length LF and EF subunits convert the current/voltage relationship of the oligomeric PA_63_ from slightly non-linear to highly rectifying and diode-like. The pattern represented by the asymmetric blockage of PA_63_ ion channel with LF and EF provided the basis for the quantitative biosensor allowing one to identify and screen small molecule compounds and antibodies that disrupt anthrax toxin components interaction. Thus, the agents that bind to either PA_63_ (but do not block the ion current) or LF/EF would inhibit the LF/EF-induced rectification of the current-voltage relationship.

Modified gramicidin was also developed as a key element of a biosensor, composed of a tethered bilayer on a solid support, the a system combining biological recognition mechanism with physical transduction [178,179,180,181]. In this application, modified GrA serves as the signal transducer: single molecule binding to its engineered domain modulates ability of the transmembrane pores to dimerize and to pass ion current. Therefore, decrease in the ion flux could be used to detect presence of the analytes and/or their identity.

### 4.3. Sequencing of Polynucleotides with αHL

**Figure 7:**
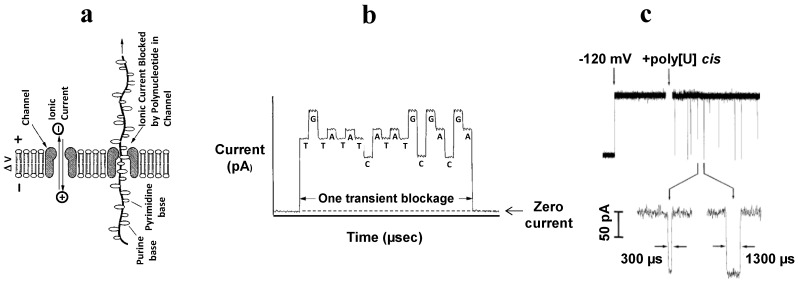
(**a**) A scheme of single-stranded DNA characterization with an ion channel. The ion current through a single unoccupied pore (illustrated by the channel at the left) is reduced as a single-stranded DNA molecule begins its passage through the pore (illustrated by the channel at the right) [182]; (**b**) Schematic representation of DNA sequencing model by the ion channel method. The individual DNA bases (G, A, T, and C) interfere sequentially and differentially with the flow of small ions through the pore which leads to the discrete conductance levels characteristic of the G, A, T, and C bases. The order of appearance of the conductance levels sequentially corresponds to the order of bases in the DNA [182]; (**c**) Oligomers of poly[U] cause transient blockades in the single αHL channel current [182]. Reprinted and modified with permission from reference [182].

An attractive idea to develop a relatively inexpensive and ultra-fast technique to decipher DNA and RNA polynucleotide sequences surfaced soon after the first reports on time-resolved polymeric molecules translocations across large toxin ion channels. The understanding that charged polymers can be successfully detected as they electrophoretically move through the appropriately narrow aperture of a biological nanopore under externally applied voltage had immediately provoked great interest to this field. The original US patent-awarded concept of the single-molecule sequencer by George Church, David Deamer, Daniel Branton, Richard Baldarelli, and John Kasianowicz [182] describes a nanoscopic channel in an artificial membrane separating two liquid-filled compartments, one of which contains the analyzed polymeric molecule, composed of sequential monomeric residues (Figure 7).

The method is based on detection of changes in the ion current through a single nanopore in the presence of a single polymer molecule that directionally moves, one monomer at a time, through the pore from one electrolyte compartment to another under the influence of an externally applied electric field. At the same time, the different passing polymer units are expected to generate different displacement of the small ions from the pore, and, therefore quite distinct current blockages (Figure 7b). The proposed principle of ion-channel assisted sensing resembles the resistive-pulse Coulter counter method, which detects and counts particles passing through a capillary connecting two electrolyte solutions [27]. The channel-based sequencer idea, due to its apparent simplicity and exciting perspectives of the fast and effective nucleic acid sequencing, has attracted multiple research groups to the nanopore-based sequencing field. This idea, if workable, would allow for scanning of polynucleotides with a µs per-base rate, making rapid sequencing of a 6 × 10^9^ base pairs of individual genome an achievable task. Moreover, in the originally proposed scheme, the nanopore sequencer idea did not require additional DNA amplification, hybridization and extensive processing, and, therefore, was expected to bring a tremendous improvement in clinical diagnostics and general research.

The early proof-of-principle experiments were performed using the wild-type αHL channel and RNA and single stranded DNA homopolymers, which were believed to be able to pass through the pore. The ion current blockades appeared immediately after polymers such as polyadenylic acid or polyuridylic acid were added to one side of a lipid bilayer containing a single αHL channel (Figure 7c). To be detected, the current-interruption events required at least 100 mV of applied voltage, positive on the side of the membrane opposite to the nucleic acid addition. These events were from hundreds of µs to several ms in duration. No channel blockage events were recorded when the polarity was reversed. This fact indicates that anionic nucleic acid molecules are driven through the channel electrophoretically [137]. As expected from the steric parameters of the αHL pore, the limiting aperture is 1.5 nm in diameter [78], which allows for a successful passage of only single-stranded DNA molecules, as shown by the quantitative PCR [137]. The additional studies produced even more encouraging results, showing that structurally specific homopolymers of RNA can be detected with reliably measurable differences in both amplitude and duration of the current through the αHL pore [183]. Thus, polyA produced blockades of ~85%, and polyC of ~95%. Dramatic differences were also observed in the average velocity with which the nucleotides traversed the pore at the same applied voltage. PolyC, for instance, passed through at 3 µs per nucleotide and polyA’s rates were 20 µs per nucleotide.

The concept of protein-pore based nucleotide sequencing has encountered many technical problems and pitfalls, even though all the steps of the original proposal, except for the reliable resolution of single nucleotide bases, had been successfully achieved [184,185,186]. To achieve a single-base resolution, ionic current through a pore should be affected by exactly one nucleotide at a time, while for αHL it was found that the pore volume can simultaneously accommodate up to 10–15 nucleotides [187,188]. Interaction of the electric field in the entrance of the pore with the entering nucleotide bases also affects the passing current [189,190]. Similarly, formation of the intra-chain intermediate structures in the passing nucleotide may produce blockage irregularities [184,191]. The sufficient rate of one nucleotide per second translocation, needed for a reliable readout, cannot be achieved at the moment, as a nucleotide is moving too fast under the applied electric field [137,192]. Besides, the temporal resolution of the pore current is highly dependent on the random motion of the polynucleotide and on non-specific interactions within the pore [186,188,193]; as a result, the translocation times for two identical molecules can differ by two orders of magnitude. 

Despite all these obstacles, great progress in this field had been achieved. The sequence of polynucleotides is now approached not only with the use of ionic channels of biological origin, such as α-hemolysin toxin and bacterial porin MspA [194], but also with engineered nanopores made in various synthetic materials, e.g., silicon nitride [186,195]. These studies are not reviewed here and their description is provided elsewhere [185,186,195,196]. 

**Figure 8:**
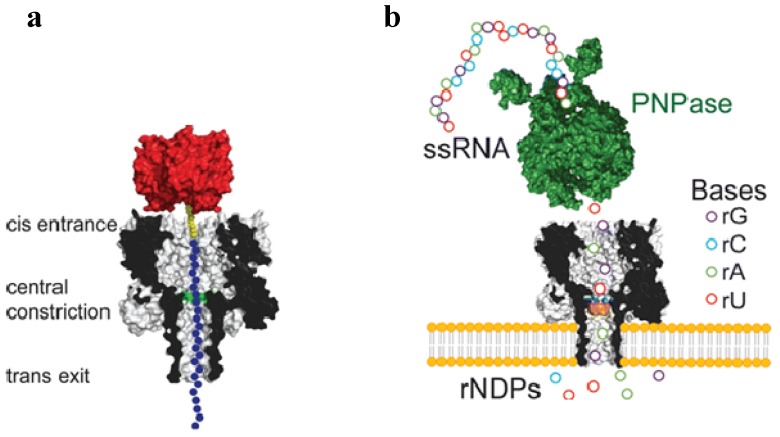
A αHL nanopore can be used to detect the single nucleotides in single-stranded DNA or RNA molecules. (**a**) A homopolymeric DNA oligonucleotide (blue circles) can be immobilized inside the αHL pore with a biotin (yellow)/streptavidin (red) linkage [199]; (**b**) Detection of the individual nucleotides cleaved from a single stranded RNA by polynucleotide phosphorylase [205]. Schematic representation of RNA oligonucleotide (circles) digested by polynucleotide phosphorylase (PNPase, green), one base at a time. The unbound nucleotides (rNDPs) are detected by the mutant M113R αHL (mutation highlighted in blue) pore equipped with a cyclodextrin adapter (orange). Reprinted with permission from references [199,205].

The task of individual polynucleotide bases recognition with αHL can be approached using different strategies. One technique focuses on slowing down the overall transport of a polynuceotide chain across the pore, so only one nucleotide in the chain is affecting the pore current. For instance, the DNA pseudorotaxane species that carry a hairpin structure at one end can be trapped in the process of translocation through the channel [197]. Reproducible current blockages are observed for various nucleotides positioned in the “scanning” constriction of the αHL pore. Similarly, the polynucleotide immobilization in the pore has been achieved by attaching streptavidin and biotin linkers to the scanned molecule [198,199]. The use of immobilized DNA allowed identification of the two specific regions within the lumen of αHL that could distinguish individual bases in polynucleotide (Figure 8a).

Another approach employs both rotoxan-additions into DNA chain and specific enzyme complexes (DNA polymerase) that slowly feed a DNA molecule into a pore in a ratchet-like motion, providing an improved signal-to-noise ratio [200,201,202,203]. The engineered pores of αHL modified to accommodate a specifically designed molecular adaptor of cyclodextrin also allowed for an improved sensitivity of this pore complex for passing individual nucleotides [199,204]. A combinational method was recently suggested [205]. In particular, the αHL pore, equipped with the permanently attached cyclodextrin adapter was able to electrically recognize single ribonucleoside diphosphates as they were processively cleaved from the analyzed polymer chain by the channel-attached enzyme, processive exoribonuclease (Figure 8b).

## 5. Channel-Forming Bacterial Toxins to Investigate Protein and Other Macromolecule Transport

Macromolecule transport across bilayer membranes is one of the most fundamental and complex life phenomena. In a recently published book [206], Muthukumar describes the process of macromolecule translocation as an ubiquitous phenomenon where electrically charged polymer molecules move to and from a highly crowded cell environment to make as a thick “Coulomb soup”. Comprehensive understanding of principles and regularities involved in the macromolecule transport is lacking and often requires investigation of an isolated translocation process *in vitro* since many biological translocation processes are too phenomenologically rich and complex for a direct *in vivo* investigation [206]. One of the powerful methods to probe polymer transport* in vitro* involves single or multi-molecule nanopore-based translocation experiments performed in the model bilayer lipid membranes. In a number of these studies, channel-forming bacterial toxins were used to exploit the pore-mediated uptake of natural and foreign polymer substrates. To a large degree, these studies were inspired by the successes in the nanopore-based nucleic acid sequencing research that we described above. However, protein translocation studies are believed to be somewhat more complicated [207]. In contrast to the linear homogeneously charged single-stranded DNA homopolymers, proteins contain multiple positively or negatively charged residues and polar and hydrophobic side chains and, therefore, are charged non-homogeneously. Besides, large polypeptides are usually folded into different structures and exist in a variety of transition states and conformations. Therefore, one of the most critical steps for the protein to navigate through the narrow biological pores would be the necessity to undergo substantial conformational transitions, including unfolding. Despite the complexity of the systems, several groups have provided essential insight into protein translocation processes using the model lipid bilayer techniques.

### 5.1. Studying Polymer and Protein Transport with aHL

The main body of publications on polymer and protein translocations investigated with the resistive-pulse sensing approach uses single αHL channels, wild type or variants, reconstituted into the artificial bilayer lipid membranes. In 1992, Krasilnikov and colleagues developed an elegant approach to estimate the effective radius of an ion channel based on the conductivity measurements performed in the presence and in the absence of water-soluble polymers, polyethylene glycols (PEGs) of different molecular weights [208]. The method was later use to determine the geometry of different ion channels, including the pores of αHL [71] and PA_63_ [209], discussed here. The approach was further refined to allow not only for determining the size of channel openings but also to detect the presence, size, and apparent localization of structural constrictions inside the pore [71]. The method was based on the effect of asymmetrical addition of PEG on the channel conductance.

An approach for covalent attachment of the single PEG molecules within the lumen of the αHL pore has been described [210]. When the polymer was functionalized by a covalently attached biotin molecule, the tethered polymer was detected on both the *cis* and *trans* sides of the membrane using genetically engineered streptavidin mutants with a weakened binding affinity [211]. Such a covalently attached and functionalized “moving arm” is designed to move across the bilayer from one opening of the pore to another and could be used as a sensor element for detecting protein analytes. Interestingly, closing linear PEG molecules into a circular “crown” substantially modifies their dynamics in the αHL pore [212]. In contrast to the linear PEGs, the blockage efficiency of the crown exhibits a strong dependence on both the sign and magnitude of the applied voltage (voltage asymmetry). The authors suggest that the charged rigid crown/K^+^ complex is responsible for the blockage. The closing of a linear polymer molecule into a circle changes its pore translocation because the complex of this cyclic molecule with a K^+^ ion faces an effective barrier in the channel. The capture and release of single PEG molecules by the αHL channel were detected as time-resolved reversible current-fluctuation events when extreme KCl concentrations (4 M) were used [213]. Remarkably, the capture on-rate, or fluctuation frequency, decreased monotonically with the PEG size, whereas the residence time of the polymer inside the channel showed a crossover behavior, initially increasing but then decreasing with molecular weight of the polymers. The authors suggest that the αHL channel is not spacious enough to accommodate larger PEG molecules and that the out-of-pore portion of the polymer molecule acts as an entropic spring pulling on the trapped part and thereby decreasing the residence time of the proteins in the channel.

Furthermore, the αHL variants carrying different point cysteine mutations were probed with small water-soluble sulfhydryl-specific reagents of different charge and mass [74,214]. These polymers bind to the cysteine side chains exposed into the aqueous phase in the αHL lumen by forming disulfide linkages at different locations. This allowed one to determine the location of a constriction at the midpoint of the pore lumen [74]. The results also suggested that the net charge of an αHL channel lumen defines the weak anionic selectivity of the channel and that the charge at the pore entrances determines the shape of the conductance-voltage dependencies [214]. Muthukumar’s group investigated the molecular mechanisms responsible for the two-level ionic current blockages produced by single poly(styrene sulfonate) molecules passing a single αHL pore under an electric field [215]. The authors showed that only the current reduction sub-events with deep current blockages were successful translocations. However, changing the pH on the *trans*-side of the pore allowed for tuning the effective charge of the β-barrel and for dramatic increase in frequency of the successful translocation events. The authors also developed a stochastic theory with a pairwise free energy profile with polymer/pore interaction, that was qualitatively consistent with the experimental results [215]. A capture of single molecules of sodium poly(styrene sulfonate) by αHL was also studied under varying transmembrane voltage, pH, and salt concentration asymmetry conditions [216]. The study reports that the electrophoretic capture of single polymer molecules by a αHL pore under salt concentration gradients is controlled by a combination of pore/polymer electrostatic interaction and induced drift force due to the salt concentration asymmetry. Besides, at higher pH, the polymer/pore interaction is repulsive and lowering the *cis* compartment salt concentration increases the effect. At lower pH, the polymer/pore interaction becomes attractive and the effect is stronger at lower *cis* compartment salt concentrations. The authors propose a hypothesis of an antagonistic/synergistic coupling between the pore/polymer interaction and induced drift by salt concentration asymmetry [216].

The interactions between the αHL’s β-barrel pore and positively charged signal α-helical peptides were also examined [138]. The transport of the specifically designed peptides with a central lysine residue within the repeat unit (Ac-(AAKAA)_m_Y-NH_2_, *m* = 2–7) was investigated with a focus on the contribution of peptide charge and length to the free energy barrier for the translocation. The authors found that the kinetic parameters of the peptide/pore binding reaction were strongly dependent on transmembrane voltage and peptide length. In particular, a rise in event frequency with the increasing of the applied transmembrane voltage and a reduction in event frequency for longer peptides were reported. The collagen-like peptides existing as mixtures of single, double, and triple helices were also distinguished and identified by comparing their characteristic translocation parameters through αHL [217]. αHL, together with aerolysin of *Aeromonas hydrophila*, was used to analyze transport of several α-helical peptides of the sequence fluorenylmethoxycarbonyl (Fmoc)-D_n_A_x_K_y_ [218]. A net negative charge of these peptides has allowed them to be driven through the pores by the applied voltage.

Several studies were designed to use biological nanopores to probe protein folding. Thus, Auvray and collaborators investigated the transport of partially folded and unfolded maltose-binding proteins through αHL under conditions that favored protein denaturation [219]. Movileanu and collaborators reported related effects when polypeptides with various β-hairpin structures produced longer ion current blockages by comparison with no-hairpin containing peptides [220]. The unfolding of the protein was shown to be important in facilitating its translocation through αHL pore, and, apparently, is the rate-limiting step for nanopore-mediated protein translocation in many systems. More recently, a technique that allowed proteins to be unfolded for processive translocation was described [221]. The authors investigate controlled unfolding and translocation of proteins through αHL using the protein unfoldase ClpX, which is a component of the ClpXP proteasome-like complex responsible for the targeted degradation of numerous protein substrates in *E. coli* and other organisms. ClpX acts by forming a homohexameric ring that uses ATP hydrolysis to unfold and translocate proteins through its central pore and generates sufficient mechanical force to denature stable protein folds. To examine co-translational unfolding of individual protein molecules, the protein substrates were tagged with oligonucleotides to enable potential-driven unidirectional movement through αHL [222]. The kinetics of the co-translocational unfolding of thioredoxin, which is a model protein of 108 amino acids was then tested at the single-molecule single αHL level. The study revealed a four-step mechanism that includes two separate unfolding events with a detectable, partly unfolded intermediate. A molecular description for each step is provided [222]. Interestingly the authors suggest that the general mechanism for the co-translocational protein unfolding that they describe is broadly similar the one proposed by Krantz and colleagues for the PA_63_-mediated uptake of lethal toxin of anthrax [223] (discussed in detail in the Section 5.2). The thioredoxin translocation through αHL was recently presented as proof-of-principle system for detection of protein phosphorylation [224].

Another important factor in protein transport is the ability of the protein to interact with the binding sites within the pore lumen. To understand how this interaction alters the underlying kinetics of polypeptide translocation, αHL variants with acidic binding sites composed of rings of negatively charged aspartic acid residues placed at the strategic positions within the β-barrel were engineered [225]. These mutations resulted in a significantly enhanced transport of cationic polypeptides across the membrane. For instance, when the entry and exit of the β-barrel were modified with the electrostatic binding sites, both the on- and off-rate of the polypeptide/channel binding reaction were significantly increased because of decrease in the free energy barrier for translocation. At the same time, hydrophobic polypeptides faced a greater energetic barrier and translocated at a considerably lower rate compared with the positively charged hydrophilic polypeptides [225]. To determine the sensitivity of the single-molecule pore recordings to the changes in biophysical features of the pore lumen or the interacting polypeptide, Mohammad and Movileanu used electrical recordings on a single αHL pore probed with short (~25 residues) cationic polypeptides and small folded pb_2_-Ba proteins consisted of positively charged precytochrome b_2_ fragments fused into the small (~110 residues) ribonuclease barnase [226]. The αHL pore contained negatively charged electrostatic traps and the kinetics of the polypeptide/pore interaction was shown to be dependent on the trap location. At the same time, the positive charge carried by the pb2-Ba proteins was not sufficient to allow the protein to undergo a substantial partition into the pore lumen. The authors followed these studies [225,226] with a simple model to describe nanopore-mediated translocation of the semi-flexible polypeptides, whose contour length is comparable with the length of the protein pore [227]. The impact of charge reversals of the αHL’s residues was also investigated with the pore variants where lysines were replaced with aspartic acids [228]. The study showed that charge-reversal mutations that disrupted ion-pair interactions on the *cis* opening of the β-barrel produced rather quiet electrical signals with reduced single-pore conductance, whereas disruption of an ion-pair interaction on the solvent-exposed *trans* opening of the β-barrel led to a significant gating activity, larger amplitude, and frequent current fluctuations in the pore. At the same time, the combination of the two charge-reversal mutations resulted in the irreversible collapse of the β-barrel detected as a large-amplitude permanent blockage of the channel current. The authors suggest that these distant charge reversals are energetically coupled and have different impacts on the ionic transport, conductance, and probability of the open state of the αHL channel [228]. The model allowed for calculation of the relative and absolute exit frequencies of the short cationic polypeptides through the *cis* and *trans* openings of the pore and for quantitative assessment of the polypeptide translocation kinetics. The kinetics of peptide/pore interactions was also investigated with peptides containing mainly aromatic amino acids in αHL pores engineered with aromatic binding sites [229]. The authors reported that with an increase in the peptide length, both the event mean dwell time and the amplitude of current blockage increases whereas the events frequency shows only slow increase, indicating that the binding affinities of the peptides are mainly dependent on the off-rate constants rather than on the on-rate constants. Besides, with more aromatic binding sites engineered in the pore lumen, a stronger binding affinity between peptides and the pore was detected [229].

**Figure 9:**
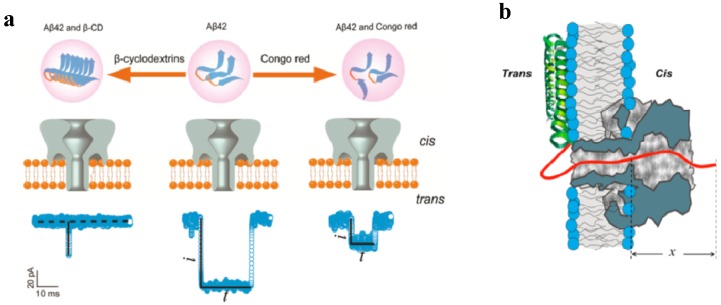
Detecting intrinsically disordered amyloid peptides, Ab42 [231] and α-synuclein [232] with αHL nanopore. (**a**) Aβ42 in the presence and absence of β-cyclodextrin (Aβ42-CD) or Congo Red (Aβ42-CR) added from the *cis-*side chamber (*top*). Representation of the blockage behavior of the translocation event of Aβ42-CR (*bottom*); “i” is equal to the difference between the open pore current and the average amplitude of the blockage current, “t” is the duration time of the blockage [231]; (**b**) Schematic model of α-synuclein interaction with the αHL channel pore [232]. While the membrane-bound helical part of α-synuclein stays on one side of the membrane, the highly negatively charged C-terminal tail of the protein (*red/solid line*) enters the αHL pore from the *trans*-side and goes past the channel constriction under the externally applied electric field. Reprinted with permission from references [231,232].

Nanopore analysis was also suggested for studying the misfolding of certain proteins, for instance amyloids, α-synucleins and prions [230,231]. The αHL pores were used to probe the different aggregation transition of β-Amyloid 42 (Aβ42) (Figure 9a) [231].

Aβ42 is the predominant form of the amyloid peptide, which is found in the plaques of the brains of Alzheimer’s patients and is an abundant component in amyloid aggregates. The peptide was studied in the presence of β-cyclodextrin, which promotes Aβ42 aggregation, and in the presence of Congo red, which inhibits the aggregation. These induced differences in the Aβ42 aggregation states were analyzed by monitoring the corresponding characteristic blockage events where β-cyclodextrin and Congo red were used to promote or inhibit Aβ42 aggregation, respectively. More recently, αHL was utilized to investigate the elongation of wild type α-synuclein and A53T α-synuclein monomers [230,233]. α-synuclein is a neuronal water-soluble protein involved in the etiology of Parkinson disease and other neurodegenerative dementias through formation of the α-synuclein fibrils. Gurnev *et al.*, observed 95% blockage of the current by α-synuclein when the compound was added from the membrane side where the shorter stem part of the channel was exposed [232] (Figure 9b). The applied potential was more negative on the side of α-synuclein addition. The binding reaction on-rate was reported to depend strongly on the membrane lipid composition. In particular, in diphytanoylphosphocholine membranes α-synuclein binding was about two orders of magnitude stronger compared with membranes made with dioleyl lipids. The turnover dependence of the dwell time on the applied transmembrane voltage strongly suggested the α-synuclein translocation. Besides, when the unstructured C-terminal domain of α-synuclein had the last 25 amino-acid residues, including nine carboxylates, removed, the compound’s binding affinity dramatically decreased. At the same time, measurements of the binding/translocation reaction parameters in 4 M KCl solutions showed impressive increase in the pore-protein attraction, possibly reflecting the osmotic and crowding effects in α-synuclein/pore interaction. 

Investigation of αHL pores for the stochastic sensing applications often involves substantial reengineering of the wild type channel. Thus, a new type of stochastic sensor based on a αHL pore modified with an aptamer was recently reported [234]. The aptamer was bound to the channel by hybridization to an oligonucleotide that is attached covalently to a single cysteine residue through a disulfide bond near an entrance of the pore. In this way, the oligonucleotide acts as an adapter to which various nucleic acid ligands can be coupled by duplex formation. The authors demonstrate that thrombin was interacting with a 15-mer DNA aptamer at nM concentrations forming a cation-stabilized quadruplex. 

### 5.2. Studying Protein Transport with PA_63_

**Figure 10:**
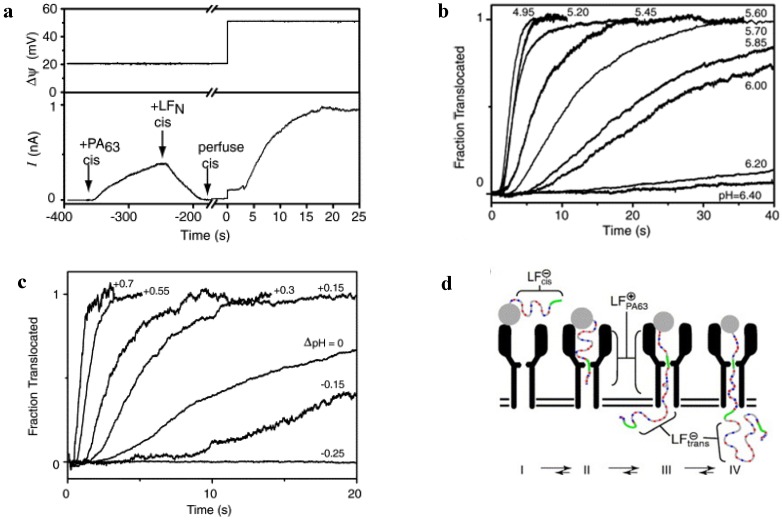
The interaction of LF_N_ with the PA_63_ multichannel membrane [239]. (**a**) After the macroscopic PA_63_-induced current had reached a steady state at +20 mV, ~3 nM LF_N_ was added to the *cis* compartment of the chamber, which resulted in a rapid fall in current. The *cis* compartment was then perfused of unbound LF_N_. At time zero, the voltage was increased to +50 mV, and LF_N_ translocation kinetics through the PA_63_ channels was indicated by the increase in conductance *vs.* time; (**b**) Kinetic transients for LF_N_ translocation at +60 mV and the indicated symmetrical pH values; (**c**) The rate of LF_N_ translocation is controlled by the magnitude and sign of the transmembrane ΔpH (pH_trans_ > pH_cis_); (**d**) A schematic illustration of the tandem Brownian ratchet translocation mechanism shows a model of several partially unfolded intermediates of LF_N_ during translocation [239]. The scheme demonstrates how the hydrophobic, ϕ-clamp ratchet and the protonation-state ratchet may work together facilitating translocation of LF_N_. Reprinted with permission from reference [239].

The substantial progress in understanding the physical principles governing anthrax toxin uptake is due to the pioneering lipid bilayer measurements performed by Finkelstein and Collier’s groups and, more recently, by Krantz’s group (reviewed in refs. [100,235,236]). The very first study investigating protein transport using the anthrax toxin was designed to answer the following two questions: (1) can LF and EF be translocated across a planar bilayer membranes in the absence of any cellular components; and (2) does the PA_63_ channel act as a conduit for this process [117]? Earlier studies with single-chain diphtheria (DT) and botulinum toxins (BT) showed that the A portion of the protein can be transported across a planar lipid membrane without the aid of any cellular components and, therefore, all of the translocation machinery for DT and BT is built into their B fragments [237,238]. At the same time, binary anthrax toxin has a significant advantage over the single-chain AB-type DT and BT toxins [117]. In particular, the channel incorporation and protein translocation events are separated in binary toxins, meaning that in the model studies of multiple or single PA_63_ channels could be reconstituted first and LF and EF added later. The authors believe that, if under these conditions translocation is captured, then the channel is not just a “discarded wrapper” [117]. An electrophysiological system for studying translocation across planar bilayer membrane is shown in Figure 10.

In contrast to the majority of publications where protein translocation through αHL was investigated, the authors chose to focus on the multi PA_63_ channel membranes. In a typical experiment, the pre-activated PA_63_ was added to the *cis* compartments of the bilayer chamber at 20 mV *cis* side positive transmembrane voltage until channel formation reached steady state, which was indicated by stabilization of the ion current [117,239]. The substrates (LF_N_, LF, EF or their variants) were then added to the *cis* compartment at the concentration that allows blockage of 95% of current. The unbound substrate was removed by perfusion from the *cis* compartment solution. The protein translocation leading to channel lumen unblocking was recorded as the increase in conductance *vs.* time. This process was triggered by either increase in the applied voltage to Δψ ≥ 40 mV (Figure 10b) or by creating pH gradient, pH_trans_ > pH_cis_ (Figure 10c) [117,239,240]. At the same time, the ΔpH that the authors used to mimic a proton gradient across an endosomal membrane was more efficient in stimulating protein transport, allowing translocation not only LF_N_ but also full-length LF and EF. The conductance rise was S-shaped, and its rate increased systematically both with increasing of Δψ and ΔpH. With the minimum PA_63_-binding translocation component domain of LF, LF_N,_ it was shown that, under small Δψ, the N terminus of bound LF_N_ enters the oligomeric channel and initiates the threading of the substrate across the bilayer membrane [116].

In order to identify residues in the PA_63_ lumen important for the channel-mediated transport of a substrate, the authors used cysteine-scanning mutagenesis coupled to [2-(trimethylammonium ethyl]methanethiosulfonate (MTS-ET) modifications [241]. The channels were the most significantly affected by Phe427/Cys427 modifications, and Phe427 (named ϕ-clamp is the most hydrophobic residue in the otherwise hydrophilic pore lining [118]. In particular, when the bulky 427 phenylalanine residue was replaced by the small alanine, the single-channel conductance expectably increased but the rate of LF_N_ translocation reduced by a factor of six [239]. Based on the fact that the ϕ-clamp mutation blocked LF translocation in cell assays [242] or significantly slowed it *in vitro* [118], a chaperone model was suggested in which the ϕ-clamp’s phenyl rings directly interacted with the translocating polypeptide. According to the model [118], the ϕ-clamp recognizes substrates through the hydrophobic effects, enhanced by aromatic-aromatic, π-π, and cation-π interactions, and stabilizes the fragments it an unfolded state. This study for the first time provided evidence that PA_63_ was not a passive tunnel through which proteins electrophorese but rather an active player in facilitating the transport of a substrate molecule. At the same time, the lumen of a 14-strand β-barrel PA_63_ is believed to be as narrow as 12–15 Å [101,174,243], which would allow the pore to accommodate secondary structure only as large as an α-helix [244]. Therefore, translocation through PA_63_ would require the enzymatic substrates to unfold. If so, what are the factors that cause the folded LF and EF with tertiary structures to unfold? The authors show that one critical factor is low endosomal pH. Thus, the acidic conditions in the endosome are sufficient to destabilize the native structure of LF_N_ and EF_N_ proteins [244]. *In vitro* experiments have also revealed that the translocation process is driven by *cis* positive voltages [117] and that the PA_63_ channel is selective to cations [175]. Paradoxically, LF_N_ bears a net negative charge (about six negative charges) even at pH values as low as 5.5 [240]. To explain this finding, the authors developed a model describing PA_63_ channel as a protein translocase rather than an unselective long tunnel. The easiest way for the substrates to achieve a net positive charge, which is thermodynamically and electrostatically required for the translocation, would be to get their aspartic and glutamic residues neutralized, for instance protonated [239]. Under ΔpH, the substrate molecule would get protons from the *cis* compartment solution (or an endosome) and discharge them into the *trans* compartment solution (or cytosol) following translocation. At the same time, the portion of LF/EF located inside the pore lumen would carry a net positive charge and PA_63_ would work as a proton-protein symporter [119].

To address the fundamental question of the translocation driving force, the authors developed a novel charge state-dependent Brownian-ratchet model for the ΔpH-driven translocation, which is based on the chemical asymmetry created by ΔpH [239]. The probability of an aspartate or glutamate being in the protonated form is greater in the more acidic *cis* side compared to the more basic *trans* side, and, therefore, the rate of their entry is higher from the *cis* side than that from the *trans* side [240]. After the protonated aspartate and glutamate groups reach the cytosolic part of the membrane, they deprotonate, becoming negatively charged again, resulting in the electrostatic repulsion of the negatively charged chain fragment from the channel lined with the negatively charged residues. This electrostatic repulsion drives the translocation per se and enforces its directionality [100,239]. Therefore, the random Brownian motion of the substrate inside PA_63_ is biased toward the more basic *trans* compartment. Based on the experimental evidence, the authors suggest that the PA_63_ symporter achieves protein translocation using a tandem of two synergistic Brownian ratchets: the ϕ-clamp ratchet, promoting the substrate unfolding, and the charge-state ratchet, which biases the entry rates of the substrates into the pore (Figure 10d).

Remarkably, further experiments with planar lipid bilayers have imparted support to this model using essentially non-titratable negatively charged SO_3_^−^ groups introduced at most positions in LF_N_ [119]. The voltage-driven translocation of the resulting LF_N_ variants was dramatically reduced and the ϕ-clamp was determined as a significant factor in the exclusion of SO_3_^−^ from the channel. In another study, semisynthetic variants of LF_N_ (12-263) in which selected acidic residues were replaced with the unnatural amino acid, cysteic acid, were examined [245]. The cysteic acid has a negatively charged side chain protonated only at pH values below the physiological range. Depending on the number of acidic residues replaced, transport of these mutants was either significantly suppressed or completely inhibited, whereas their binding and channel-blocking properties were comparable with those of wild type LF_N_. To determine if the substrate’s secondary structure is preserved during the pore-facilitated translocation, a method of trapping the polypeptide chain of the translocating protein within a pore was developed [121]. In order to determine the minimum number of residues that could traverse the PA_63_ oligomeric pore, the authors attached biotin to the N terminus of LF_N_ and used molecular stoppers at the different positions. The *trans*-side streptavidin addition was used to determine whether the N terminus has reached the *trans* compartment solution. If the N terminus-stopper distance was long enough to allow for LF_N_ to appear from the pore, streptavidin was able to bind the biotin. Otherwise, no biotin binding was recorded. The authors showed that an LF_N_ polypeptide chain adopts a fully extended conformation, because it is being translocated through the channel’s stem.

A typical characteristic of LF_N_ translocation is its non-exponential S-shaped kinetics. At the same time, in most of the experiments the substrate translocation was investigated under conditions where two or three LF_N_ molecules were bound to PA_63_ channels. To understand whether the S-shaped kinetics is an intrinsic characteristic of translocation kinetics or a consequence of the translocation in a tandem of two or three LF_N_ molecules, a kinetic analysis of protein transport via the macroscopic and single-channel PA_63_ channels was performed [120]. The study showed that even with one LF_N_ bound to PA_63_, the translocation kinetics is S-shaped, being, however, slower, with more than one LF_N_ bound. The authors also propose a simple drift-diffusion model of LF_N_ transport, where LF_N_ is represented as a charged rod that moves subject to both Brownian motion and an applied electric field across the membrane [120].

**Figure 11:**
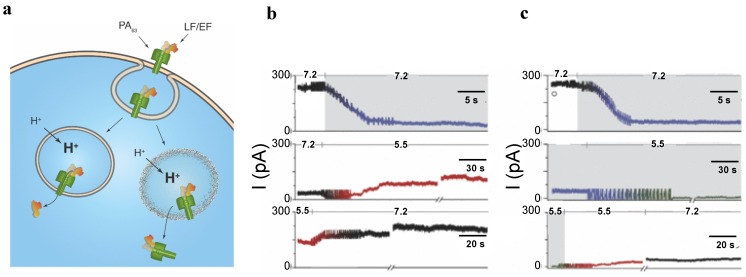
(**a**) A schematic illustration of two proposed mechanisms for PA_63_ channel-mediated lethal factor (LF) and edema factor (EF) transport into the cytoplasm. The channel-mediated translocation model suggests that LF and EF pass through the pore [118,239]. The recent membrane rupturing model suggests that the enzymatically active anthrax toxin complexes (namely, LF or EF bound to the PA_63_ channel) rupture membranes [18]. Time course of the PA_63_ channel conductance with (**b**) LF removed before or (**c**) maintained at 1 nM during *cis*-side acidification. (*Top rows*) ~60 PA_63_ channels were reconstituted into a planar bilayer membrane at pH*cis*|*trans* 7.2|7.2 (black) and, 1 nM LF was added to the *cis* chamber (blue). (*Middle rows*) The pH*cis*|*trans* 5.5|7.2 gradient was formed by perfusing the *cis* chamber with pH 5.5 buffer that contained either [LF] = 0 (red) or [LF] = 1 nM (green). (*Bottom rows*) The neutral pH condition (pH*cis*|*trans* 7.2|7.2) (black) was restored by perfusing the *cis* chamber with pH 7.2 buffer. If LF was present, the *cis* chamber was first perfused with pH 5.5 buffer (red) then pH 7.2 buffer (black). Reprinted with permission from reference [18].

A dramatically different model of anthrax toxin transmembrane uptake was recently suggested by Kasianowicz and colleagues [18]. The new model suggests that instead of LF and EF being threaded through the pore, anthrax toxin complexes (*i.e.*, LF or EF bound to the PA_63_ channel) rupture membranes (Figure 11).

Kasianowicz *et al.*, question the translocation model, noting that the *in vitro* pH gradient formation used in the previous translocation studies is precisely opposite to what occurs *in vivo* [18]. The translocation, manifested by PA_63_ ion current recovery (Figure 10), was observed either in static low pH conditions on both sites of the membrane (mostly with LF_N_) or starting with the symmetrical acid conditions and forming a pH gradient by raising the *trans* solution pH (both with LF_N_, EF_N_, LF, and EF). In the real *in vivo* systems, PA_63_ oligomeric prepore complexes would first bind the LF and/or EF subunits on the cell surface, then undergo endocytosis, and, after that, under pH_endosome_ < pH_cytosol_ conditions, the prepore/pore transition would occur. To support the alternative anthrax toxin uptake model, the authors show that anthrax toxin complexes can rupture both the planar bilayer and the droplet membranes [18]. Moreover, the transmembrane pH gradients alter the ion conducting properties of the PA_63_ pores and LF/PA_63_ interaction. In particular, under conditions that mimic those across the endosomal membrane, the strength of LF/PA_63_ interaction in the absence of LF excess in the solution is relatively weak. However the LF binding is irreversible when LF is present in bulk during acidification [18]. To understand the binding of LF/EF to the channel, the authors designed an experiment starting from essentially different experimental conditions (Figure 11b) compared with those used for the translocation experiments. PA_63_ channels were incorporated into the planar bilayer membranes under symmetrical pH_cis_ = pH_trans_ = 7.2 conditions (Figure 11b, top). Following channel formation, LF was added to the *cis* compartment solution and the remaining unbound LF was flushed from the chamber. Lowering the pH *cis* solution to 5.5 led to a slow current increase (Figure 11b, middle) explained by dissociation of LF from the channel; the current continued to increase when pH was increased to 7.2 (Figure 11b, bottom). About 90% of current recovery was observed after 30 min [18]. To show that acidification promotes the LF dissociation from the channel, the authors repeated the experiment (Figure 11c) maintaining the bulk *cis*-solution LF concentration at constant during the acidification. For that purpose, the *cis* solution was perfused with the LF containing pH 5.5 buffer (Figure 11c, middle). After that, the solution was additionally perfused with LF-less pH 5.5 buffer (Figure 11c, middle). The described procedure led to only minor increase in the current even after pH was return to 7.2 (Figure 11c, bottom). Thus, about 90 min after returning to initial pH_cis_ = pH_trans_ = 7.2 conditions, only 17% of the initial PA_63_ current was recorded, indicating strong, nearly irreversible LF/PA_63_ interaction when [LF]_bulk_ = 1 nM during acidification. The authors also showed that when LF or EF were in the bulk during acidification of the *cis* side, the formation of the essentially irreversibly bound LF:PA_63_ and EF:PA_63_ complexes led to membrane rupture.

In addition, the article contains interesting suggestions about a possible alternative mechanism of the anthrax toxin uptake, which the authors support with carefully designed experiments and detailed discussion [18]. We believe these data must be addressed in the future. On the other hand, it is important to emphasize that the earlier studies showed nearly ideal positive correlation between the translocation rate measured for different LF, EF, and PA_63_ variants *in vitro* and cytotoxicity of the complexes in cell assays and *in vivo*. Moreover, a variety of available and rationally designed small molecule and polyvalent compounds aimed to specifically obstruct the PA_63_’s translocation pathway were shown to be very effective in inhibiting anthrax toxin [111,118,163,165,167,176,246]. The positive correlation between binding activity of these blockers to the PA_63_ channel* in vitro* and their protective action *in vivo* was reported. The dominant-negative PA mutants that co-assemble with the wild-type PA_63_ and block its ability to translocate the LF and EF components have been also described [247,248,249,250]. 

## 6. Channel-Forming Bacterial Toxins for Cancer Therapy

Many traditional anticancer agents, while being highly effective, also show their well-known toxic properties toward normal fast proliferating cells. These drugs were often discovered in cellular screens of extracts from natural sources, or in *in vivo* screens using a leukemic P388 mouse model, and entered the clinical studies and market before the exact mechanism of their action was understood [251]. In contrast, emergent next-generation strategies of the anticancer drug discovery focus on targeted therapies, where the agents are designed rationally to target the unique features of malignant cells. Thus, cancer cells overexpress specific tumor antigens, carbohydrate structures, and growth factor receptors on their surface or they express cancer specific proteases [252]. Targeting these factors is a widespread strategy under development for a selective killing of cancer cell using small molecules, monoclonal antibodies, modified bacterial, plant and fungal toxins, viral nanoparticles, and any other inhibitors, which follow the principles required to selectively destroy cancer cells.

The bacterial toxins are naturally cytotoxic: a property that makes them attractive to use in the targeted therapies. Thus, only one molecule of diphtheria toxin fragment A introduced into a cell can kill the cell [253]. The major task is to direct the cell-binding properties of the bacterial toxins for selective action on cancer cells while minimizing their ability to destroy healthy cells. At first, the targeted toxin research focused on design of so-called “immunotoxins”, molecules consisting of a protein toxin fused to a tumor cell-specific antibody or antibody fragments. This approach afterwards expanded to include other target-specific ligands, such as growth factors or cytokines. These proteins are often referred to as “targeted toxins” (TTs). Pseudomonas exotoxin A of *Pseudomonas aeruginosa* and diphtheria toxin of *Corynebacterium diphtheria*, which enzymatically inhibit protein synthesis, are commonly used for the immunotoxin construction. The *Clostridium* and *Bacillus* binary toxins are also considered excellent candidates for the targeted toxin design. Being composed of two (or three in the case of anthrax toxin) nontoxic proteins, the binary toxins offer a significant advantage over the single-chain AB-type toxins, because activities of their binding and enzymatic components can be redirected independently towards targeted cancer cells. The membrane-perforating bacterial toxins and pore-forming antimicrobial peptides have also served as excellent tools for the targeted toxin research. For the specific aim of the current review, here we highlight examples of the therapeutic strategies that are focused on a targeted modification of the membrane-perforating bacterial toxins, such as αHL, and on the channel-forming components of the binary toxins, such as PA_63_. For examples of other targeted toxin applications and for a general overview of the field, we direct the reader to a series of recent reviews [254,255,256,257,258,259,260,261,262,263,264].

Anthrax toxin is an excellent choice for targeted toxin research [265]. The *Bacillus anthracis* infections are rare and currently only a limited number of people have been immunized against anthrax. According to the CDC, only laboratory workers who work with anthrax, some veterinarians, and some members of the US army are routinely vaccinated. Therefore, in contrast to diphtheria toxin (childhood immunization) and Pseudomonas exotoxin (earlier infections), most people lack pre-existing immunity for anthrax. Besides, the tripartite nature of the anthrax toxin offers a variety of tuning strategies for targeted modifications of the non-linked toxin components. Moreover, substantial recent progress made in the understanding of anthrax toxin protein structures and uptake mechanisms facilitates design of tailored antitumor agents.

**Figure 12:**
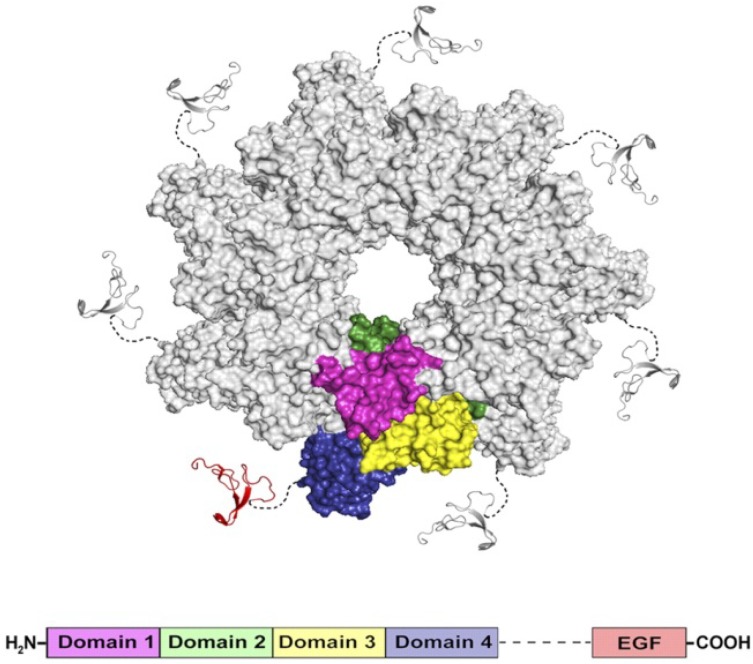
The receptor-based approach to re-engineer PA by disrupting the native receptor-binding function of the toxin and specific linking of the mutated protein to a heterologous receptor-binding protein [268]. Composite representation of the heptameric prepore formed by PA_63_ (PDB: 1TZO) with EGF (PDB: 1JL9) linked to the C-terminus. An axial view of the heptameric prepore is shown, with domains 1, 2, 3, and 4 in a single subunit of PA_63_ colored magenta, green, gold, and purple, respectively. EGF is in red. Broken lines represent an 8-amino-acid linker (SPGHKTQP) connecting the N terminus of EGF to the C terminus of PA_63_. Reprinted with permission from reference [268].

Targeted anthrax toxin research progresses in at least three different directions, which are often combined. First, because PA binding to the CMG2 and TEM8 cell surface receptors is the first event in the multistep anthrax toxin’s intracellular transit, redirecting the protein towards alternate tumor cell specific receptors was investigated. To demonstrate the feasibility of this approach, it was shown that tailor-made PA can be targeted towards the human p62 (c-Myc)-specific hybridoma cell line 9E10 [266,267]. The C-terminus of wild-type PA was fused to the amino acid residues 410–419 of the human p62^c−Myc^ epitope via a (G_2_S)_2_ linker; ac-Myc IgG was proven to act as alternate receptor. The PA-c-Myc fusion protein was shown to kill RAW cells, which do not express the c-Myc receptor. The addition of the c-Myc epitope to the C-terminus of PA did not interfere with the ability of the fusion protein to bind to the PA TEM8 receptor. Therefore, the presence of competitive inhibitor PA SNKEΔFF (a non-toxic receptor-binding mutant) and the fusion protein FP59 (amino acid residues 1–254 of LF and the ADP-ribosylation domain of Pseudomonas exotoxin) was required to fully protect 9E10 cells from a challenge with PA-c-Myc and FP59. Another receptor-redirected approach is to disrupt the native receptor-binding function of the toxin and then to specifically link the mutated protein to a heterologous receptor-binding protein. Recently, the native receptor-binding activity of PA was ablated by introducing N682A and D683A mutations in domain 4 [268]. The resulting C terminus of the mutated protein was then fused to one of two heterologous receptor-binding proteins: human epidermal growth factor or the receptor-binding domain of diphtheria toxin (Figure 12). The resulting PA variants mediated the cell entry of the active components of the toxin. The developed approach was used to redirect toxin action to cells bearing the HER2 receptor [269,270].

**Figure 13:**
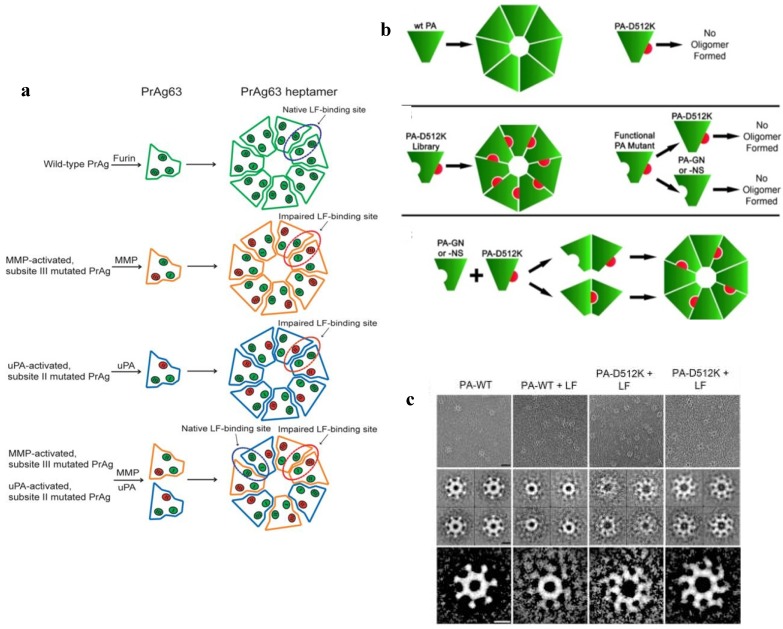
(**a**) Scheme for reengineering anthrax toxin protective antigen (PA) to modify its action towards two distinct proteolytic activities overexpressed by the cancer cells [281]; (**b**) Scheme for discovery of PA mutants that exclusively form octamers [282]; (**c**) EM images of heptameric and octameric PA species [282]. Reprinted with permission from references [281,282].

The unique requirement that certain toxins have to be active on the cell surface of the targeted cell provides strategies to tailor these toxins to make them dependent on the cancer cell-specific proteases. Therefore, a second approach to re-engineered PA is to replace the furin cleavage site with cleavage sites for proteases that are present on the surface of cancer cells. For the broad-spectrum TTs, it is practical to focus on the cell surface-associated proteases that are overexpressed in a variety of tumor tissues and tumor cell lines, namely on the matrix metalloproteases (MMPs) and urokinase plasminogen activator (uPA, not to be confused with PA, the binding/translocation subunit of anthrax toxin). To examine the role of MMPs in the design of TTs, two mutated PA proteins were constructed in which the furin protease recognition site RKKR was replaced by the GPLGMLSQ (PA-L1) and GPLGLWAQ (PA-L2) sequences [271]. These fusion proteins were designed to be susceptible to cleavage by MMP-2, MMP-9 and MT1-MMP and were rapidly and selectively activated on the surface of MMP-overexpressing cancel cells. The resulting PA-L1 and PA-L2 oligomers were used to internalize a recombinant FP59 fusion protein and these combinations were shown to be selectively toxic to MMP-overexpressing tumor cells, which included human fibrosarcoma, breast cancer, melanoma and thyroid carcinoma [267,271,272,273,274,275]. To examine the role of uPA in the design of TTs, a set of mutated PA proteins in which the furin activation site was replaced by uPA recognition sequences was also constructed [276]. The uPA substrate sequences, GSGRSA and GSGKSA were used to replace the furin RKKR sequence in PA, which yielded mutated PA proteins, PA-U2 and PA-U3 that were efficiently activated by uPA. PA-U2/FP59 was later investigated as a wide-range, highly selective, and highly potent chimeric toxin that specifically targets uPA-expressing tumors, independently of their tissue origin [277,278,279,280]. In an elegant study, Leppla and colleagues reengineered PA-constructing mutants with mutations affecting different LF-binding subsites and containing either uPA or MMP cleavage sites [281] (Figure 13a).

These proteins contained additional mutations so that PA-U2 and PA-L1 monomeric subunits had to be adjacent in an oligomer to form native LF binding sites. As a result, individually the constructed PA variants showed decreased toxicity due to the impaired LF binding; however, when administered together to uPA and MMP overexpressing cancer cells formed functional LF-binding heteroheptamers. The mixture of these two mutants was highly effective *in vivo* to treat diverse aggressive transplanted tumors. In this study, the authors established proof-of-principle that anthrax toxin can be re-engineered in a way so that its cytotoxicity relies on two distinct proteolytic activities overexpressed by the cancer cell [281]. This strategy was further examined in a study where the therapeutic window of the anthrax toxin-based TTs was enlarged with PA variants rationally designed to selectively and exclusively form oligomers [282] (Figure 13b).

The idea of this design was based on a finding made by Krantz and colleagues that PA, which for a long time was believed to form heptameric complexes, is also able to form octamers, and they are functional [107]. Moreover, conditions under which octameric oligomerization predominates were determined and heptamer/octamer structural comparison showed that there are two orientations of the receptor-binding PA domain 4, which alternate in the octamer. The molecular determinants that influence the stoichiometry of PA complexes were identified, e.g., the relative proportion of PA heptamers and octamers could be controlled by tethering domain 4 to domain 2 with two different length cross-links [283]. By screening a highly directed library of PA mutants, Leppla and colleagues identified variants that complemented each other to form octamers exclusively [282]. The authors started with the PA mutant D512K, which is incapable of forming oligomers (Figure 13b, top), and prepared a <50,000-members library of PA variants that have the D512K substitution together with random mutations in several residues on the complementary face of PA_63_ within the oligomers (Figure 13b, middle). The library was screened for regaining of oligomer-forming ability and then, after the sought variants were identified, D512K and new complementary mutations were placed into two separate PA proteins. As a result, the authors achieved formation of oligomers through the use of the two unique interfaces (wild type and mutated), which resulted in formation of even-numbered oligomers (Figure 13b, bottom), predominately octamers (Figure 13c). In particular, the authors focused on the transformants containing substitutions at residues 245 and 252. By combining PA-D512K with either PA K245G/R252N (abbreviated as PA-GN) or PA K245N/R252S (abbreviated as PA-NS) generated high toxicity comparable to that of wild type PA both *in vitro* and *in vivo*. However these variant were non-toxic when used individually [282]. 

The third strategy is based on the fact that the PA_63_-mediated protein uptake machinery is effective enough to deliver multiple engineered variants of the native anthrax toxin LF and EF components. To increase the therapeutic benefit of the TTs, the native LF and EF were replaced with a variety of fusion proteins containing the PA_63_ binding N-terminal domain of LF (LF_N_) or EF (EF_N_) and different toxophores (the so called “cut and paste” approach [265]). The resulting chimeric variants included LF_N_ fused to Pseudomonas exotoxin A enzymatic domain [284], Shiga toxin enzymatic domain, diphtheria toxin A chain [285,286], tetanus toxin light chain [287], *Clostridia difficile* B toxin glycosylating domain [288], *Haemophilus ducreyi* cytolethal distending toxin B subunit [289], Bcl-XL protein [290], ricin toxin A chain [269], flagellin [291], and beta-lactamase [292,293]. Many of these heterologous proteins have been successfully delivered by PA_63_ (native or mutated) into the cytosol. However, some proteins, apparently those that were not able to adopt a partially or fully unfolded state at the acidic endosomal pH, have failed [267,294]. Another factor that can limit the potency of TTs is the stability of the enzymatic domains in the cytosol [295]. Recently, this issue has been examined with LF_N_-PEIII variants constructed with insertion of a ubiquitin domain (wild type and mutated) between the targeting domain (LF_N_) and the catalytic “payload” (PEIII) [295]. The variants were designed to address the previously described bias against the presence of lysine residues in enzymatic domains of several AB-type bacterial toxins [296]. Thus, the catalytic domains of cholera toxin, *E. coli* heat-labile toxin, Shiga-like toxin, and ricin have strong bias towards Arg relative to Lys. This characteristic is believed to limit the attachment of ubiquitin followed by the proteasomal degradation of the toxins [295,297]. Ubiquitin is a small eukaryotic regulatory protein, which, among its multiple functions, also has a signaling role in protein degradation. The cytosolic stability of LF and LF_N_-based chimeric proteins was shown to follow the so-called N-end rule [294,298] described by Alexander Varshavsky in 1986 [299]. In accordance with the N-end rule, the specific destabilizing N-terminal amino acid of a protein controls the effectiveness with which side chain lysine residues are ubiquitinated for the following proteasomal degradation. Ubiquitin, after binding to a target protein, can be in its turn ubiquitinated, forming polyubiquitin chains on any of the seven lysine residues within ubiquitin. This process is controlled by deubiquitinating (DUBs) enzymes that allow for ubiquitin to be released from the protein. Interestingly, LF_N_-Ub-PEIII fusion proteins, in which all seven lysines of the wild type ubiquitin were retained while the site cleaved by cytosolic DUBs was removed, were nontoxic, which the authors explain by a rapid ubiquitination and proteasomal degradation [295]. The authors also showed that the fusion protein, in which all seven lysines were substituted by arginine (Ub_Knull_), had high potency exceeding that of FP59. In general, the potency of these proteins was highly dependent on the number of lysines retained in the ubiquitin domain and on retention of the C-terminal ubiquitin sequences cleaved by DUBs [295]. The stability of LF_N_-PEIII fusion proteins was also significantly improved in a study where N-terminal amino acids of LF_N_ were mutated and all the lysines reductively dimethylated [300]. 

Besides the single chain and binary toxins, membrane-perforating bacterial toxins were also considered as potential candidates for the tailored modifications. Among the bacterial pore-forming toxins which were investigated for their cytolytic or cytocidal properties against tumorous cells were α-hemolysin of *Staphylococcus aureus* [26,301,302,303], parasporin-4 of *Bacillus thuringiensis* [304,305,306,307], listeriolysin O of *Listeria monocytogenes* [308], aerolysin of *Aeromonas hydrophila* [309,310], the S component of Panton-Valentine lekocidin (LukS-PV) of *Staphylococcus aureus* [311] and epsilon toxin of *Clostridium perfringens* [312]. In particular, Bayley and colleagues explored αHL mutants in which channel-forming activity can be triggered or switched on and off by biochemical, chemical, or physical stimuli [26,301,302]. The approach was based on earlier studies, which showed that the subunits of a functional αHL with nicks near the midpoint of a central glycine-rich loop are supported by domain-domain interactions, whereas αHL oligomers containing two truncated subunits that overlap in the central loop had greatly reduced channel-forming activity [313,314,315]. Based on these data, the group designed overlap αHL mutants that were activated when redundant amino acids in the loop were removed by proteases that inactivate the wild-type protein [301]. The authors proposed that this strategy could be used to construct proimmunolysins, variants of αHL that would be preferentially activated by the cancer cell surface proteases [301]. αHL would then perforate and kill the tumor cell or permeabilize the cell membrane for the cytotoxic drugs that have no or low permeability [303]. Thus, combinatorial mutagenesis was used to obtain αHL mutants that were rapidly and preferentially activated by cathepsin B [303]. Cathepsin B, normally an enzymatic protein, is involved in various pathologies and oncogenic processes in humans; its overexpression is correlated with invasive and metastatic phenotypes in cancer [316].

A series of tailor-made pore-forming bacterial toxins were generated for intracellular delivery of different types of macromolecules [317,318]. One of the main challenges drug designers face is poor bioavailability of the compounds as a result of poor membrane permeability. Loaded pH-sensitive listeriolysin O-containing liposomes were developed [319,320,321,322,323,324,325] and tested both *in vitro* and *in vivo* (see refs. [19,317] for details). Targeted drug delivery to generate protective antiviral immunity was also examined using the anthrax toxin [266,326,327,328]. Interestingly, gramicidin, the first marketed natural antimicrobial agent (1939) [329], was also studied for inhibition of human immunodeficiency virus (HIV) infection [330,331,332,333].

## 7. Concluding Remarks

Promising new developments in nanopore biotechnology continually emerge [334]. Because of the overwhelming number of the reports in this popular field, the current review is not exhaustive. In this article, written for a special volume on “*Intracellular Traffic and Transport of Bacterial Protein Toxins*”, we highlight many interesting applications of channel-forming bacterial toxins in small and macromolecule sensing and polymer transport. For clarity, instead of discussing all pore types previously investigated, we focus on three structurally and functionally different bacterial toxins: (1) peptide gramicidin A (GrA) of *Bacillus brevis*, which induces lesions in cell membranes forming small exclusively cation-permeable channels; (2) α-hemolysin (αHL) of *Staphylococcus aureus*, which targets the host cell membranes by forming large β-barrel pores; and (3) protective antigen (PA_63_), the pore-forming B component of the anthrax toxin, which mediates translocation of the toxin’s enzymatic components inside the target cell. There is no doubt that the number of related publications on biotechnological applications of any of these toxins far exceeds all the residual reports in the bionanopore field. At the same time, the reasons why these particular molecules were chosen for development are mani-fold. Gramicidin A can serve as an ideal single molecule on/off switch reacting to different stimuli and as a sensor for lipid membranes properties. The αHL channel is a structurally stable pore, which remains in the electrically low-noise open state over a wide range of experimental conditions, including extreme salt concentrations, pH, and temperatures. The crystal structure of αHL was solved at a 1.9-Å resolution more than 15 years ago and the protein could be genetically or chemically modified in a number of ways. αHL was recently reengineered to form a functional bottom-up dimeric pore than spans two adjacent lipid bilayers [335]. On the other hand, when αHL was so severely truncated that the protein heptamer could not span a bilayer, the channel insertions were still observed, supporting the idea that membrane proteins could stabilize the toroidal lipid pores [336]. Interestingly, the channel-forming B component of the anthrax toxin, PA_63_ is often compared with the membrane pore of αHL. This is mostly because these channels were reported to form functional mushroom-shaped heptamers. However, electrical characteristics of PA_63_ differ dramatically from those of αHL. First, despite having comparable diameters at the most narrow constrictions, the αHL conductance in 1 M KCl solution is ~5 times higher, which allows for a significant improvement of the signal-to-noise ratio at the single channel level. Second, since formation of the PA_63_ channels are triggered by the low endosomal pH, the available pH range for the molecular sensing experiments is significantly limited for subacidic pH. Finally, two types of gating were reported for the PA_63_ channels. The typical voltage gating, intrinsic to many β-barrel channels incorporated into the artificial bilayers [337,338], shows a pronounced voltage asymmetry in the case of PA_63_ pore, somewhat limiting the voltage range available for the statistically reliable measurements. More importantly, the current noise through the open PA_63_ channel is far from being electrically quiet, showing persistent 1/f fluctuations between open and closed states. Similar to αHL, PA_63_ was shown to be able to accommodate βCD molecules. However, the current blockage events were complete, which limits the possibilities to use βCD as PA_63_-modifying adapters. Therefore, it was not the PA_63_ channel of being used as a biosensor *per se*, but rather an elevated interest in the anthrax toxin and several strong research groups working in the field that advanced biotechnological applications for the anthrax toxin. Attempting to understand the anthrax toxin uptake mechanism by investigating protein transport on the one hand, and the unique ability of reengineered PA_63_ to deliver enzymatic components inside the target tumor cell on the other hand, made this “noisy” channel a nanopore of choice for different studies. Some of these reports created directions for the rational modifications of PA_63_ that could significantly alter the pore properties. For instance, F427A PA_63_ mutant was reported to have ~3-times higher conductance than the wild type pore and did not show the wild type PA_63_’s 1/f noise current fluctuations [102,118]. A number of interesting dominant negative [247,248,249,250] and predominantly octameric PA_63_ mutants was reported [282]. The anthrax toxin-neutralizing antibodies reconfiguring PA into supercomplexes, which are yet to be tested in the bilayer membranes, were also described [339]. GrA, αHL, and anthrax toxin’s channel forming component PA_63_ are available commercially and, in contrast to other toxins, for instance epsilon toxin and botulinum neurotoxin, there are no firm biosafety restrictions on the usage of small quantities of these proteins under regular laboratory conditions.

There is no doubt that the nanopore biotechnology is and will remain an exciting field of research. In the future, with the development of single-channel electrical recording techniques, X-ray crystallography, protein engineering, computational methods, and, importantly, human curiosity, we will see many interesting new pore types being explored. As an example, a group of peptide phytotoxins produced by *Pseudomonas syringae*, syringomycin E (SRE), and similar compounds were extensively studied by Schagina and colleagues [135,340]. Similarly to gramicidin A, SRE-produced pores can be used as sensitive probes of the membrane physical state, surface charge, orientation of membrane inner dipoles, and the interaction of membrane-active molecules.

A cytolytic pore-forming toxin aerolysin of *Aeromonas hydrophila* represents an interesting alterative to αHL. Recently, X-ray crystallography, cryo-EM, MD and computational modeling approaches have been used to resolve a near-atomistic structure of the aerolysin pore and to study intermediate states leading to the pore formation [341]. Both biosensing and protein translocation properties of the channel have been reported [342,343,344,345]. Another “αHL-like” oligomeric pore is formed by cholesterol-dependent *Vibrio cholerae* cytolysin [346,347,348]. Remarkably, the X-ray crystallography [349] revealed an interesting structural detail of cytolysin—a narrow constriction region formed by an unexpected aromatic tryptophan W318 ring of residues within the pore that is otherwise rich in charged amino acid residues. The authors compare this region with the ϕ-clamp of the B component of the binary bacterial toxins, such as anthrax, where it is believed to be critical for the A component’s translocation. At the same time, so far there is no evidence indicating that *Vibrio cholerae* cytolysin serves as a transmembrane protein translocase. We wonder if this structural property could be employed to mimic the Brownian ratchet mechanism of anthrax toxin translocation [239] with this significantly different protein *in vitro*.

Currently, one of the greatest problems hampering the study of polymer transport and nucleic acid pore-assisted sequencing is the high translocation rate of these molecules. Several approaches have been developed to slow down the single-molecule transport through a protein nanopore [192,350,351,352]. Thus, genetically optimized porin MspA of *Myobacterium smegmatis* is believed [19] to be a promising pore for biosensing and sequencing applications due to its conical shape providing a very narrow (~10-Å) sensing zone [194]. Interestingly, epsilon toxin of *Clostridium perfringens* (reviewed in refs. [353,354,355]) was reported to form slightly anion-selective stable low-noise pores with a single-channel conductance in the range of 440–640 pS in 1 M KCl [356,357]. The polymer-partitioning studies to access the epsilon toxin pore’s functional shape and size suggested that the channel is highly asymmetric, *i.e.*, conical with the tentative radii of openings of 0.4 and 1.0 nm on the *cis* and *trans* sides, respectively [358]. However the single channel studies on this channel are limited to three publications [356,357,358], which could be partially explained by lack of the epsilon toxin oligomer’s crystal structure and by certain CDC regulations placed on use of this category B agent.

Besides, the channel-forming B components of the clostridial binary bacterial toxins show remarkable structural and functional similarities with PA_63_ [9,318]. Moreover, PA_63_ was shown to be effective in transporting the His-tagged enzymatic C2I component of the binary C2 toxin into the cytosol [359,360]. Along with the anthrax toxin, binary clostridial ADP-ribosylating toxins have been examined for their ability to deliver heterologous catalytic domains inside the tumor cells. Many of the reported examples, which mainly consist of the chimeric toxins constructed on the basis of the active component of C2 toxin, C2I are intensively reviewed elsewhere [9,318]. More recent reports describe genetically engineered chimeric C2IN-streptavidin complexes, which were designed to delivered biotin-labeled molecules into the cytosol of diverse eukaryotic cell lines by the binding/translocation subunit of the toxin [361,362,363]. The C2-streptavidin delivery system was used to internalize biotin-labeled p53 tumor suppressor into different mammalian cell lines, including human tumor cells [364]. The direct C2IN-p53 constructs were also investigated [365]. Chemical conjugation strategies as alternatives to engineering fusion proteins have also recently been explored resulting in the assembly of C2 toxin-based Janus-like supramolecular fusion proteins based on the iminobiotin-avidin linkage responding to external stimuli, such as pH [366]. Currently, it is unclear to what extent the channel-forming B components of the clostridial binary toxins serve as active translocase of their A components, similarly to what is suggested for PA_63_. On the one hand, the preserved ϕ-clamp in position 428 was shown to be important for pore formation and for cytotoxicity and rounding up of cells by the enzymatic C2I subunits of the C2 toxin [367]. On the other hand, the host cell chaperones Hsp90 and the peptidyl-prolyl *cis/trans* isomerase cyclophilin A were reported to be critical for membrane translocation of the active moieties of clostridial C2, iota, and CDT toxins but not for LF of the anthrax toxin [368,369,370]. Provided that the corresponding bilayer measurements show successful translocation events similar to those described for the anthrax toxin, we believe these findings would add some useful arguments to the anthrax toxin uptake debate [18]. For that purpose, not only the native but also the PA_63_/C2I-His cross-combination of components could be tested.
